# A study on multi-omic oscillations in Escherichia coli metabolic networks

**DOI:** 10.1186/s12859-018-2175-5

**Published:** 2018-07-09

**Authors:** Francesco Bardozzo, Pietro Lió, Roberto Tagliaferri

**Affiliations:** 1 NeuRoNe Lab, DISA-MIS, University of Salerno, Via Giovanni Paolo II 132, Salerno, 84084 Fisciano Italy; 2 Computer Laboratory, Department of Computer Science, University of Cambridge, 15 JJ Thomson Ave, Cambridge, CB3 0FD UK

**Keywords:** Multi-omics, omic regularities, Antibiotic response, Multi-omic metabolic networks, Multi-omic motifs, E. *coli*

## Abstract

**Background:**

Two important challenges in the analysis of molecular biology information are data (multi-omic information) integration and the detection of patterns across large scale molecular networks and sequences. They are are actually coupled beause the integration of omic information may provide better means to detect multi-omic patterns that could reveal multi-scale or emerging properties at the phenotype levels.

**Results:**

Here we address the problem of integrating various types of molecular information (a large collection of gene expression and sequence data, codon usage and protein abundances) to analyse the E.*coli* metabolic response to treatments at the whole network level. Our algorithm, MORA (Multi-omic relations adjacency) is able to detect patterns which may represent metabolic network motifs at pathway and supra pathway levels which could hint at some functional role. We provide a description and insights on the algorithm by testing it on a large database of responses to antibiotics. Along with the algorithm MORA, a novel model for the analysis of oscillating multi-omics has been proposed. Interestingly, the resulting analysis suggests that some motifs reveal recurring oscillating or position variation patterns on multi-omics metabolic networks. Our framework, implemented in R, provides effective and friendly means to design intervention scenarios on real data. By analysing how multi-omics data build up multi-scale phenotypes, the software allows to compare and test metabolic models, design new pathways or redesign existing metabolic pathways and validate in silico metabolic models using nearby species.

**Conclusions:**

The integration of multi-omic data reveals that E.*coli* multi-omic metabolic networks contain position dependent and recurring patterns which could provide clues of long range correlations in the bacterial genome.

**Electronic supplementary material:**

The online version of this article (10.1186/s12859-018-2175-5) contains supplementary material, which is available to authorized users.

## Background

In the last decades, the study of E.*coli* treatment tolerance metabolic response through multi-omics is emerging as an essential part of several approaches to molecular biology, metabolic engineering and medicine [1]. Nowadays promising models on multi-omics are based on statistical methodologies [2] and, recently, on multiplex approaches [3, 4]. High-throughput omics technologies [5] enrich complex relational data structures (i.e., XML documents [6], complex networks or maps of multi-view omics [7]) and provide increasing elements for the multi-omic integration at different layers of quantitative and relational information.

In several works, the bacterial metabolic response upon perturbations is modelled through the multi-omics dynamic changes on metabolic, signalling and regulatory networks [8]. Then, the multi-omic analysis leads to several engineering and optimizations approaches [9, 10] that reveal hidden biological motifs and pattern regularities [11, 12].

The integration of single omics, even if not biologically comparable (e.g. codon usage and protein abundance), can increase the total information about the system [13]. Ishii and Tomita [14] describe in depth the concept of multi-omic spaces as a powerful data-driven approach to understanding biological processes and systems. The information elicited from specific multi-omic spaces is multi-layered and phenotypic responsive [15].

**Fig. 1:**
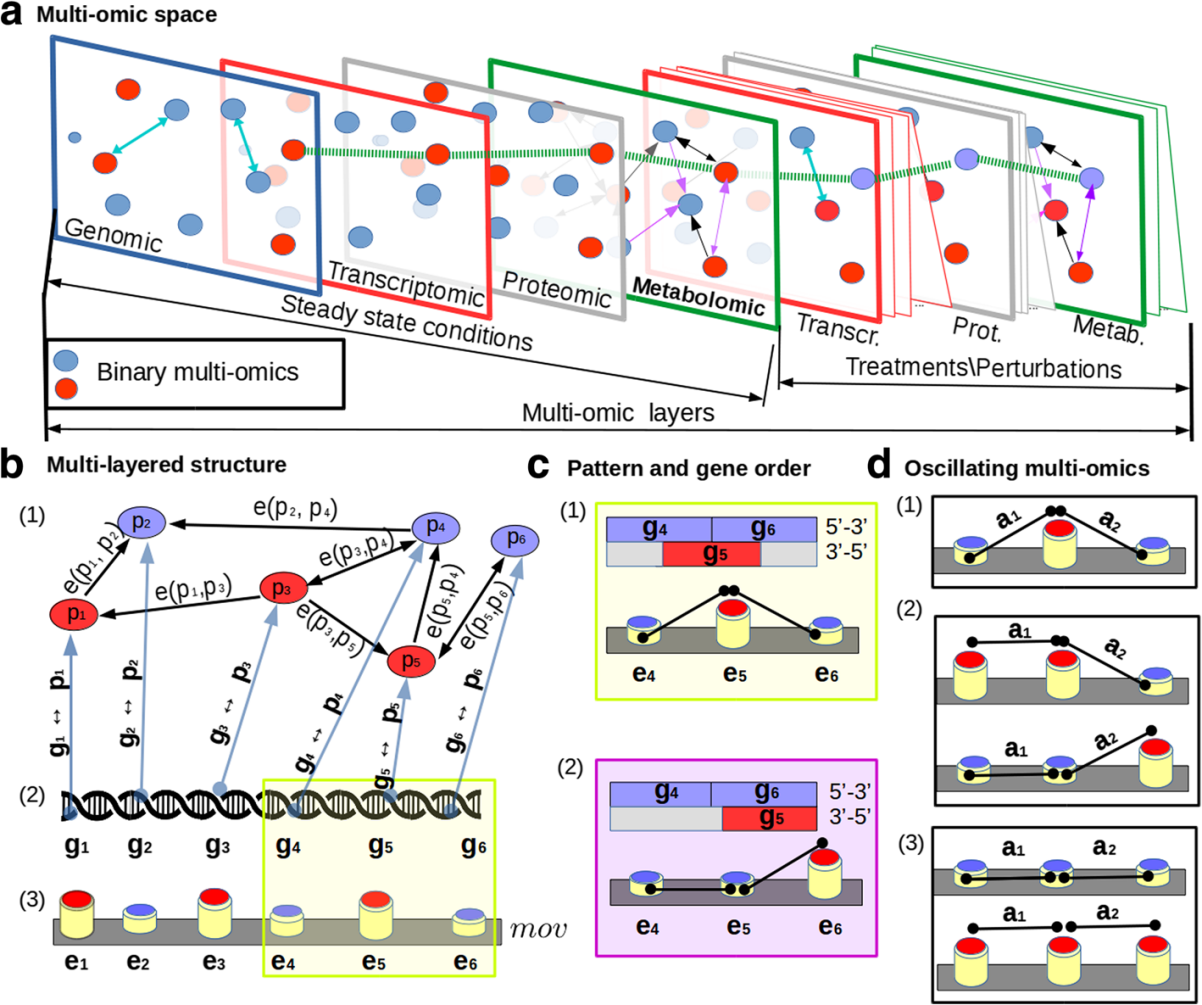
The E.*coli* multi-omic space is represented in Figure **a**: different layers represent different omics. Genomic layer (blue rectangles) presents binary discretized omic values, the same for the other layers. Multi-omics in steady state conditions could be perturbed by induced treatmens thus increasing the number of layers for each multi-omic space. Then, the number of perturbed layers depends on the number of experiment considered. Recurring multi-omic patterns motifs related with pathways are represented with multi-omic layers structure (MLS), as shown in Figure **b**. Figure **b** part *(1)* represents the j-th pathway in relation with its associated set of genes (Figure **b** part *(2)*); the resultant multi-omic pattern is shown in Figure **b** part *(3)*. The recurring multi-omic pattern is an array of pathway gene products multi-omic values arranged by the gene order. Multi-omics on the patterns are oscillating, in other words, low values follow high values and vice-versa. This feature is deeply related to the gene positions as shown in Figure **c** part *(1)* and *(2)*. Oscillating multi-omics are present in succession along the pattern as shown in *a*_1_ and *a*_2_ of Figure **d** part *(1)*. The patterns lose their oscillating features if two adjacent multi-omic values are not oscillating in an half (*a*_1_ of Figure **d** part *(2)*) or completely (*a*_1_ and *a*_2_ of Figure **d** part *(3)*)

In conclusion, identifiable multi-omic motifs could reveal the dynamical behavior of the total cellular system in standard conditions and after perturbations. Multi-omic metabolic network motifs are short recurring patterns that are presumed to have a biological function. Often they indicate sequence-specific parts of pathways with associated oscillating multi-omics. In this work, multi-omic metabolic network motifs [16] are identified and their recurring oscillating multi-omic patterns are analysed. Oscillations are defined in a binary or, at least, discrete space of features. We represented the oscillating multi-omics in two different ways: (i) as linked nodes with opposite characteristics on networks (refer to blue-red nodes of Fig. 1b (1)), (ii) as a sequence of high-low adjacent values (refer to blue-red cylinders of Fig. 1b (3)). Then, the oscillating multi-omic features on sequences and networks are linked into a multi-layer relational structure (MLS) strengthening the relations between the sequence patterns and the network motifs.

Multi-omic data integration is well documented in several works [17, 18]. In our paper we adopt noise robust techniques on up-to-date data (Additional file 1: Section S5). We will show that oscillating multi-omics are found on E.*coli* metabolic networks as motifs and on sequences as patterns.

For this reason, we introduce the MLS on which an ad hoc algorithm (MORA - multi-omic relational adjacency) finds the reciprocal influences of the neighbouring multi-omics on sequences and projected on networks. Oscillating multi-omics and their variations are helpful in the analysis of the impact of new drugs and in applications of metabolic engineering. Moreover, this work contributes to the study and to the creation of new interesting metabolic circuits based on multi-omic structural relations. Furthermore, the MORA reciprocal influences could seen as an index of the topological interplay between the gene order (considered in our sequences) and the pathways. Gene order along sequences and pathway structures are evolutionary conserved, then this index could be useful in evolutionary organism comparisons. Oscillating multi-omic motifs and patterns coupled with the MORA reciprocal influences describe in a new fashion system homeostasis processes and their regulatory functions unveiling the extent of multi-scale oscillating multi-omics and their network plasticity [19].

## Methods

The subject organism of this study is the E.*coli* K-12 MG1655 [20].

**Fig. 2:**
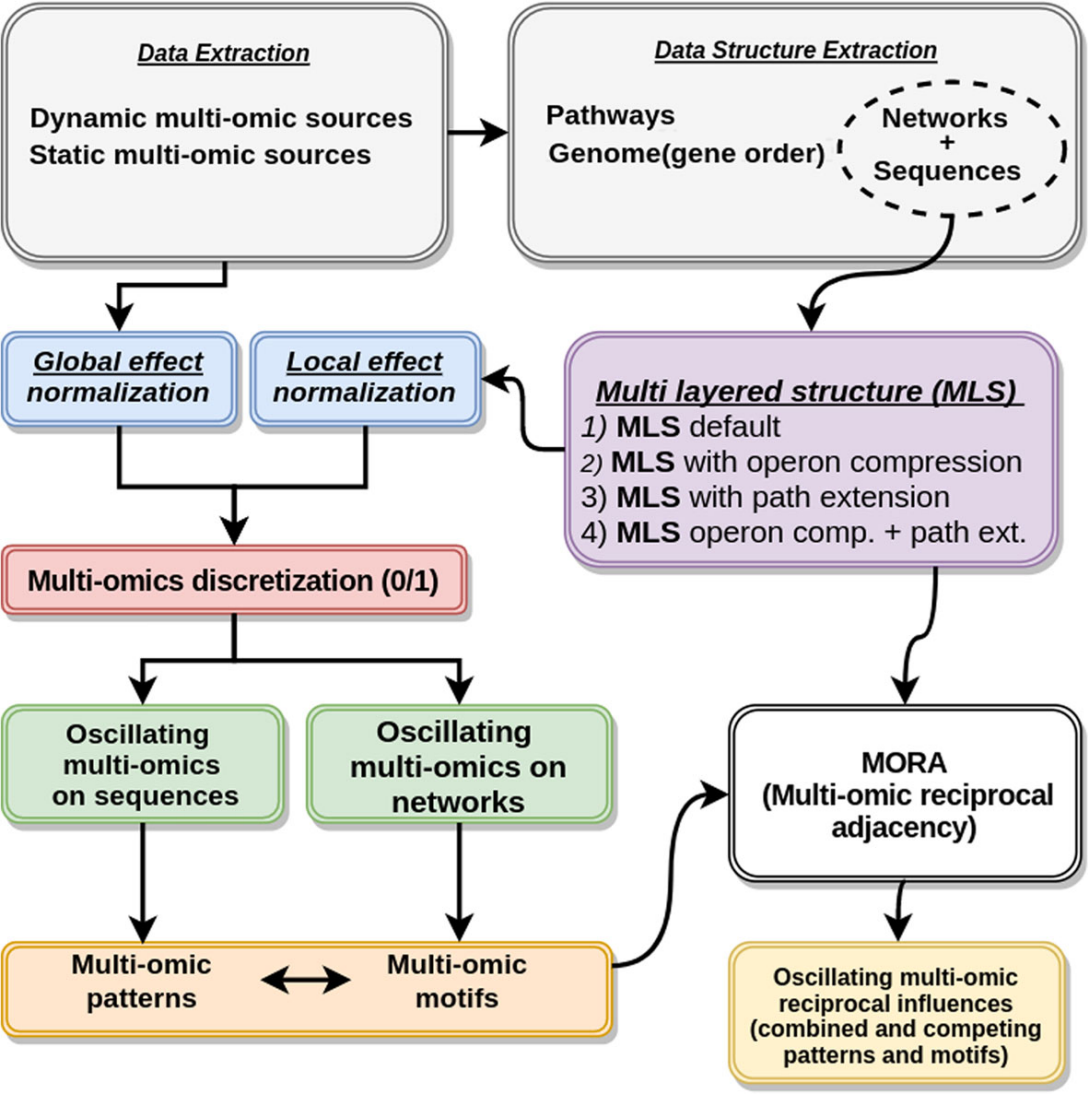
In this Figure, the whole procedure block diagram is described. Gray blocks represent the extraction of multi-omic values and structures. Then, the global and the local effects (blue blocks) are computed. The local effect depends on the type of Multi-layered structure (network + sequence) (violet block). Once the multi-omic effects are computed and normalized, then these values are discretised in 0/1. After that, the oscillation measures are computed in the respective structures (networks and sequences). The generated multi-omic patterns (from sequences) and motifs (from networks) are given in input to the algorithm MORA for the computation of their reciprocal influences. This procedure is computed in standard conditions and after perturbations obtaining combined and competing patterns/motifs

In “Definition of MLSs” section MLSs are described in detail. The global impact of antibiotics on the whole network and the local impact on pathways have been taken into account on these structures. Therefore, the multi-omic feature scaling and normalization are applied twice (please refer to “Binary discretization of multi-omics taking into account the global and the local effect” section for more detail). Multi-omics are discretized into a binary field in order to be analysed. Through the MLS it is possible to outline the relations (or reciprocal influences) of oscillating multi-omics across sequences and small networks (pathways). For this latter purpose, in “An algorithm to discover multi-omics relational adjacency (MORA)” section, the algorithm MORA is introduced. Reciprocal influences are not enough informative for understanding if the oscillating multi-omics are actual motifs of the bacterial system. Then, two models to represent the extent of oscillating multi-omic motifs/patterns as sequences (paragraph Oscillating multi-omics on patterns) and on pathways (“Oscillating multi-omics on networks” section) are introduced. A detailed description of data-sources, procedures and methods of data-integration is provided in “Data sources and multi-omics data integration” section and in the Additional file 1: Section S5. To facilitate the reader a block diagram of the overall procedure is shown in Fig. 2. We have concentrated our attention on E.*coli* organism but it is possible to extend the methodologies to other bacterial organisms. One of the most important preconditions is the availability of data: (i) the whole genome, (ii) the protein abundance, (iii) operon and protein complex information and (iv) the whole metabolic network. The most relevant bottlenecks in the preliminary data integration processes come from the availability of the protein abundance and operon information. In the PaxDB (protein abundance database) [21] the data coverage for *(i)* E.*coli* is of 98%, *(ii)* H.*pylori* is of 98%, *(iii)* B. *henselae* if of 85% and *(iv)* S.*enterica* is of 59%. Other proteobacteria have a data coverage lower than the 47%. Moreover, the operon information (DOOR DB [22]) is conspicuous only on E.*coli* (152,785 entries), instead, in the other PaxDB listed organisms is less than half or completely not numerically relevant. Nevertheless, the main lack of information, except for E.*coli*, comes from the reconstruction of the whole metabolic network. In particular, this information is important in two steps of the procedure that we will present: (1) in searching a parameter (the average path length) of the algorithm MORA, (2) in the computation of the pathways with extensions and/or operon compressions.

The most of the times incomplete metabolic networks (i.e. obtained merging only the KEGG pathways) do not present the properties of complex networks, such as the power-law degree distribution, the small world, the average path length, etc. Moreover, the power-law degree parameter *α* is important to assess if a network is biological one or not (see the *duplicaton model* of Chung et al. [23]). For this reason, as described in Additional file 1: Section S5, our integrated network is deeply studied under its biological aspects. In the domain of bacteria, for all we know, there is not another E.*coli* protein-centric network more complete than this one. For this reason, it is made available in the annexed repository.

### Definition of MLSs

In an integrated multi-omic space, as that in Fig. 1a, each omic layer is arranged on the basis of its *data-structure relations*. In the genomic layer, the genes disposed along the double strand with specific positions are transformed in a one line sequence. The gene order is considered as an organism-specific order relation (≤) and is highly conserved in duplication and during the translational processes [24]. As shown in Fig. 1b*(2)*, we described a gene relationship of the type *g*_1_≤*g*_2_≤⋯≤*g*_*n*_ for each gene *g*_*i*_. The order induced on the multi-omic sequence reproduces the gene order relation on the double strand. In particular, multi-omics are said to be adjacent with respect to the gene pairs when they are in the positions *g*_*i*_ and *g*_*i*+1_ ∀*i* ∈[1…*n*]. As it is shown in Fig. 1c*(1)(2)* *g*_4_,*g*_5_,*g*_6_ could be on the same strand or on both. As said previously, the gene reciprocal positions on the double strand are merged and represented on a line sequence. Thus, when the gene order changes in one of the two strands, then the multi-omic pattern (Fig. 1b*(3)*) changes the arrangement of its values. Indeed, in Fig. 1c*(1) and (2)* the fragment of the pattern changes because of the swap of *g*_5_ and *g*_6_.

In an integrative approach, some specific *data-structure relations* could involve more than one omic layer. For example, the proteomic and the metabolomic layers can be represented as a protein-centric network of reactions *G*(*V*,*E*) where the node-set *V* contains proteins and the edge-set *E* represents the enzymatic reactions. In the protein-centric network representation, as illustrated in Fig. 1b*(1)*, the reversible reactions are depicted as a double arrow link (⇔) and the irreversible reactions are represented as single arrow links (←*or*→) [25].

In this setting, two proteins *p*_*i*_,*p*_*j*_∈*V*(*G*)) are said to have a *strong relationship* if they are linked by an edge *e*(*p*_*i*_,*p*_*j*_) ∈*E*(*G*) or if they are the end points of an undirected shortest path that must not be longer than the average path length (APL) [26]. The proteins in a strong relationship will be the subject of thorough analysis as described in “An algorithm to discover multi-omics relational adjacency (MORA)” section.

In literature, it is proved that the gene adjacency is conserved across prokaryotes with a relevant operon architecture [27]. In particular, it has been shown that the proteins encoded by conserved adjacent genes present interactions on the metabolomic layer [28]. In presence of protein complexes, these interactions are physical, while, when dealing with anabolic and catabolic processes, they are functional. Then, the genes are positioned on DNA depending on their association to metabolic functions.

In order to model the relationship between the gene order and the pathways, the concept of MLS is introduced.

The MLS represents the pathways (Fig. 1b*(1)*) in combination with the gene order information (Fig. 1b*(2)*) studying the patterns on multi-omic sequences (Fig. 1b*(3)*). The abbreviation *mov* is used when we refer to a sequence of multi-omic values on the multi-omic sequences.

**Fig. 3:**
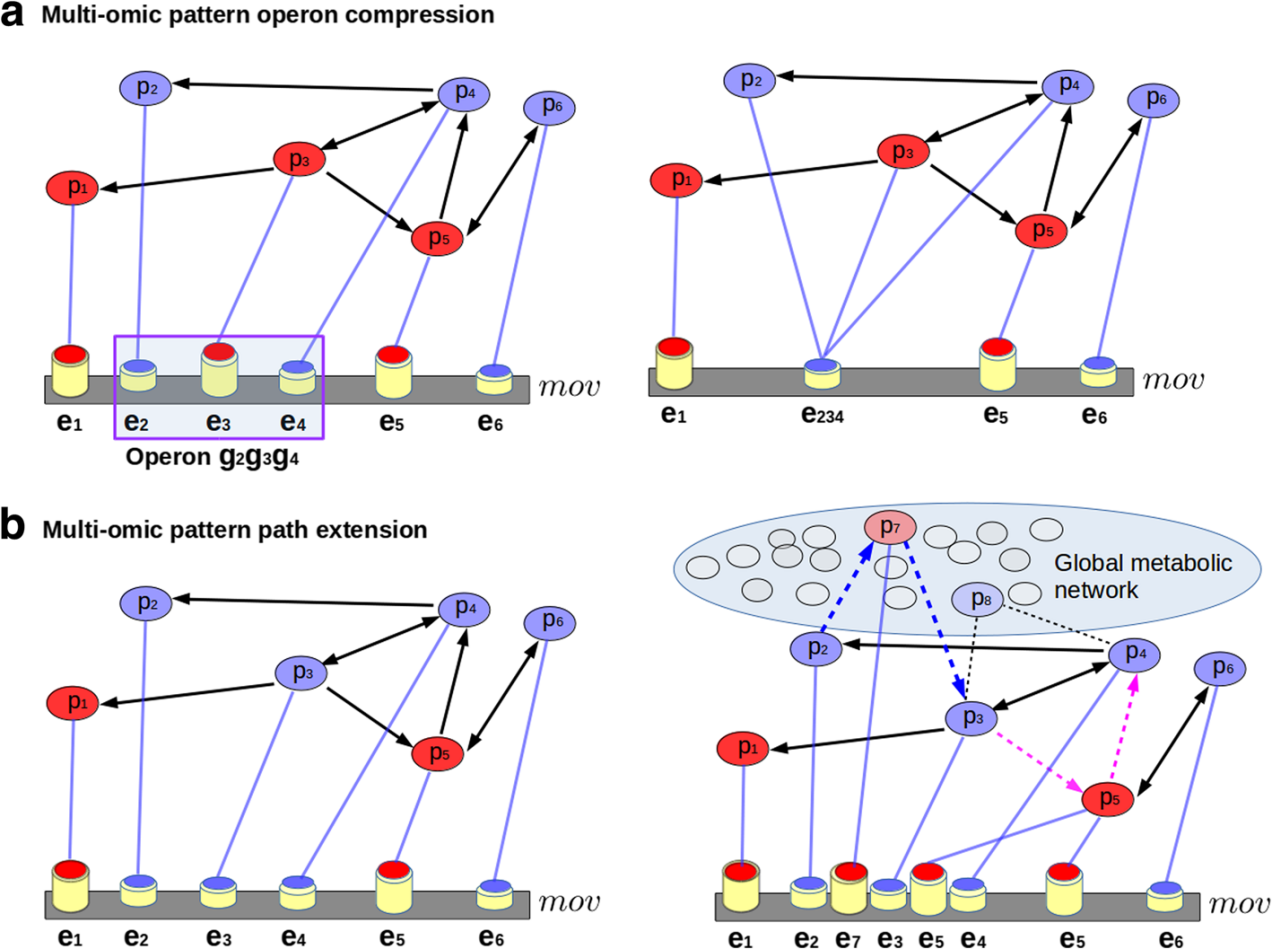
Multi-omic pattern operon compression is shown in Figure **a**. The elements that belong to the same operon (*e*_2_- *e*_3_- *e*_4_) are merged to the more frequent multi-omic value: in this case the low one (blue-head cylinder). The path extension is shown in Figure **b**. In this case, the MLS is modified searching an alternative path, on the global metabolic network, that links two nodes associated to two not oscillating pattern adjacent multi-omics (i.e the multi-omics in the positions *e*_2_- *e*_3_ and *e*_3_- *e*_4_). The multi-omic path, chosen from among all the alternative paths on the whole metabolic network, is the shortest path with the most oscillating multi-omics. (i.e in the path extension between *e*_3_- *e*_4_ is chosen the path *p*_3_- *p*_5_- *p*_4_ (violet dotted lines) and not the path *p*_3_- *p*_8_- *p*_4_

The multi-omics related to each pathway are used to build a multi-omic subspace that represents the values of each MLS. Furthermore, MLS represents the interactions *g*_*i*_⇔*p*_*i*_ ∀*i*∈*G* between the elements in different omic layers of the same subspace (Fig. 1b). Additionally, *Operon compression* (Fig. 3a) and *path extension* (Fig. 3b) are modifications of MLS, introduced to identify relevant multi-omic pattern variations. The operon compression maintains unaltered the MLS gene order. In this case, the elements that belong to the same operon (Fig. 3a *e*_2_- *e*_3_- *e*_4_) are compressed to the more frequent multi-omic. Moreover, path extension is a multi-omic pattern modification accomplished into two steps: 1) adjacent non oscillating elements on the pattern are labeled as *end nodes* of the pathway (i.e. in Fig. 3b the multi-omics in positions *e*_2_- *e*_3_ and *e*_3_- *e*_4_); 2) we search for the best oscillating shortest path that links the above end nodes.

As a result, the chosen multi-omic path is the shortest between the most oscillating multi-omics. The alternation is measured by using the score defined in Eq. 7. Then, these nodes extend the pathway and insert new multi-omics in the multi-omic sequence, breaking in some cases the gene order (Fig. 3b). Moreover, detailed aspects of oscillating multi-omics are illustrated in the following paragraphs.

### Binary discretization of multi-omics taking into account the global and the local effect

Protein abundance is a measure of the part per million quantity of the proteins inside a cell, as provided, for example, in Wang et al. [29]. Its definition and the protein abundance variation used in this paper, respectively *pa* and *pv*, can be found in Additional file 1: S5 *Section 5.0.3)*. Codon Adaptation Index (*CAI*) is an index of non-uniform codon use defined by Sharp and Li [30] (a deeper discussion is in Additional file 1: S5 *Section 5.0.1)*. All these quantities are extracted, integrated and normalized with the purpose of identifying multi-omic patterns and their *mov*.

**Fig. 4:**
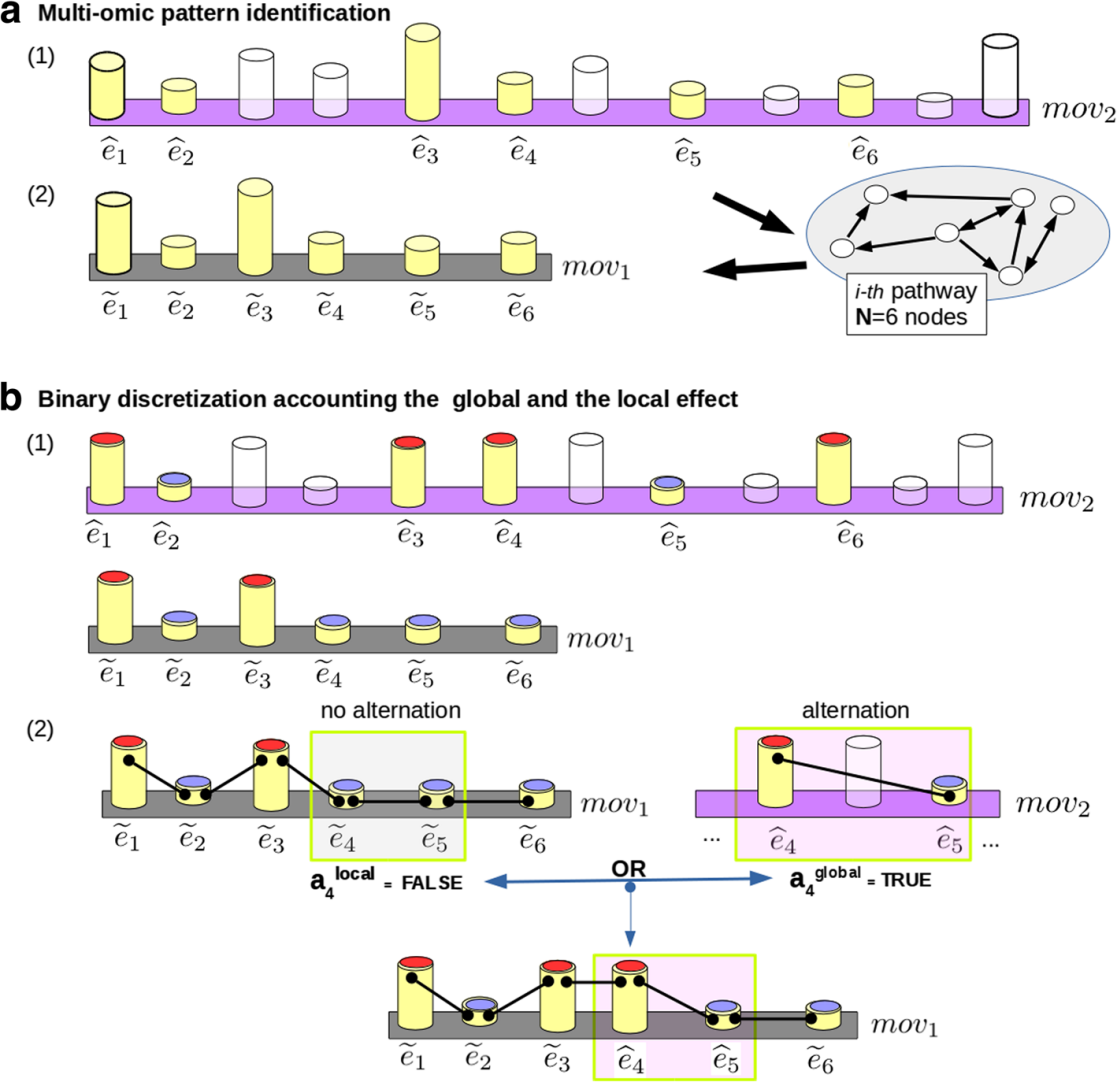
Figure 4 (**a**) part *(1)*: Multi-omics are normalized considering the complete multi-omic space. Figure 4 (**a**) part *(2)*: For each recurring multi-omic pattern, the multi-omics are normalized considering a small sample filtered from the multi-omic space by a specific pathway of *N* elements. Then, the global effect vector *mov*_2_ and the local effect vector *mov*_1_ are obtained: both the vectors have the same lenght but different multi-omic normalized values. Figure 4**b** part *(1)*: The vectors of the global effect (pink) and the local effect (gray) are binary discretized. Figure 4**b** part *(2)*: In order to consider the global response to treatments, the missing *mov*_1_ oscillations are substituted with the *mov*_2_ oscillations (if they are present). In this example the 4-th oscillation is FALSE ($a_{1}^{local}$) on the local vector and is present ($a_{1}^{global} = TRUE$) on the global vector. Then, the local effect is updated with the information of the multi-omics that come from the global effect. This procedure is done in steady state conditions and after perturbed by treatments multi-omic values

Then, the zero-mean unit-variance normalization is applied twice: i) the first time it is computed on the complete data set, i.e. considering the whole organism (see Fig. 4a*(1)*). The second time it is computed considering the same omics but on a small sample filtered from the multi-omic space by a specific pathway of *N* elements (see also Fig. 4a*(2)*). Then, a vector of numbers called *local effect*, is obtained for each pathway: ${mov}_{1} = (\widetilde {e}_{1}, \widetilde {e}_{2} \dots, \widetilde {e}_{N})$. The same elements of the multi-omic space are selected from the normalized complete data set getting a vector of real numbers called *global effect*: ${mov}_{2} = (\widehat {e}_{1}, \widehat {e}_{2} \dots, \widehat {e}_{N})$ (see Fig. 4). Both the vectors represent the same elements and have the same length of *N*. The normalization of the omics is described in the following Eq. 1:

1 $$  {}\overline{pvi}_{j}= \frac{{pv}_{j} - \mu_{pv}}{\sqrt{\sigma^{2}_{pv}}},\\ \overline{pa}_{j}= \frac{{pa}_{j} - \mu_{pa}}{\sqrt{\sigma^{2}_{pa}}},\\ \overline{cai}_{j} = \frac{{CAI}_{j} - \mu_{CAI}}{\sqrt{\sigma^{2}_{CAI}}},  $$

where *μ* is the mean and *σ* is the standard deviation. Then, the local effect (*mov*_1_) and the global effect (*mov*_2_) in steady state (see Eq. 2) and after the t-th treatment (see Eq. 3) vectors are discretized into two classes: 0 and 1 (Fig. 4b*(1)* red-head and blue-head cylinders, respectively). Binary discretized multi-omics in steady-state conditions are obtained considering $\overline {{pa}_{j}}$ and $ \overline {cai}$ ∀*j*∈1,...,*N* and *i*=1,2 as in the following Eq. 2:

2 $$ \begin{aligned} {mov}_{i_{j}} = \left\{ \begin{array}{ll} 1 & if \ \ \frac{\overline{pa}_{j} \ + \ \overline{cai}_{j}}{2} \geq 0\\ \ 0 & if \ \ \frac{\overline{pa}_{j} \ + \ \overline{cai}_{j}}{2} < 0 \end{array}\right. \end{aligned}  $$

Instead, the binary discretized multi-omics perturbed by treatments are obtained considering the protein variation for the t-th treatment $\overline {pv}^{t}$ and $ \overline {cai}$ ∀*j*∈1..*N* and *i*=1,2 as in Eq. 3

3 $$ \begin{aligned} mov^{t}_{i_{j}} = \left\{ \begin{array}{ll} 1 & if \ \ \frac{\overline{pv}^{t}_{j} \ + \ \overline{cai}_{j}}{2} \geq 0\\ \ 0 & if \ \ \frac{\overline{pv}^{t}_{j} \ + \ \overline{cai}_{j}}{2} < 0. \end{array}\right. \end{aligned}  $$

In some cases, the *mov*_1_$\left (\text {or}~mov^{t}_{1}\right)$ binary discretization is not enough sensitive to discover an alternation. Therefore, in the same positions it is possible to find oscillating multi-omics on *mov*_2_ (or $mov^{t}_{2}$) and not on *mov*_1_. If it happens then the missing oscillating multi-omics on *mov*_1_ are substituted with the oscillating multi-omics of *mov*_2_. In this way, it is possible to combine in a binary field the information about the system global response and the pathway local response to antibiotics. Formally, as shown in Fig. 4b*(2)*, the i-th local $\left (a_{i}^{local}\right)$ and global $\left (a_{i}^{global}\right)$ oscillations are taken in OR. The binary *mov* (or *mov*^*t*^) resulting from the multi-omic pattern is projected on the associated pathway.

Normalization processes are suited to deal with the assumption of independent and dependent systematic biases [31]. Moreover, the scale on which data should be included in these processes (global versus local scale) has an extensive application to high-throughput omics analysis [32]. In particular, local normalization has the advantage of correcting systematic stress response bias within small groups of multi-omics. Then, it is possible to account inconsistencies among the multi-omics once they are discretized in a binary field. The local variabilities in standard and perturbed measurement conditions could be more relevant with respect to global normalization even if they could be affected by noise. This is also the reason because we adopted noise robust techniques of data-integration as described in “Data sources and multi-omics data integration” section. However, the local normalization process may over fit the data, reducing accuracy, especially, i.e., if the multi-omics are integrated from genes that are not randomly spotted on the array [33] and subject to local and global responses determined by the interaction of global and local regulatory mechanisms, such the E.*coli* oxygen response [34]. For these reasons, it is more accurate to combine the information that comes from the global normalization with the local one.

### An algorithm to discover multi-omics relational adjacency (MORA)

**Fig. 5:**
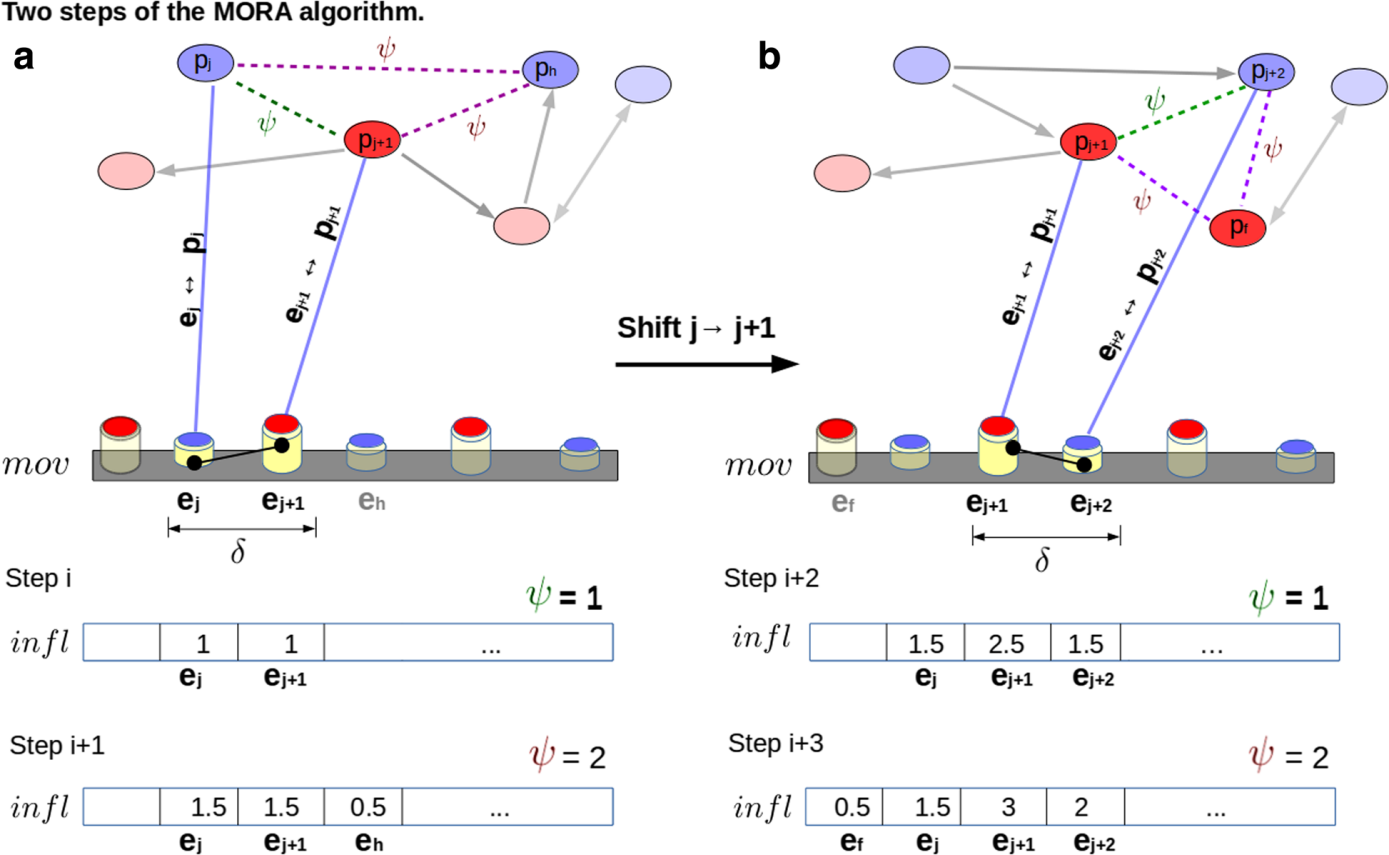
Two steps of the MORA algorithm. In the first step (part **a**), given the average path length (APL), MORA searches the shortest paths between the two adjacent multi-omics ***e***_***j***_ and ***e***_j+1_. of length: *ψ*∈[1, ⌈*APL*⌉]. The green dotted line indicates paths of length *ψ*=1 and the magenta dotted lines represents paths of length *ψ*=1, MORA does not searches a path of length 3 (which would imply *ψ*=3) because we supposed that the APL = 2. The algorithm then updates the weight vector *infl* and moves on the next pattern positions where searches for the next adjacent multi-omics (***e***_***j***_**+1** and ***e***_***h*=**j+2_). In the second step (part **b**) MORA evaluates the shortest paths for *ψ*=1 and 2. The array of the weights *infl* is updated, as shown in the step *i* to *i*+3, according to the algorithm

MORA is a search algorithm that weights the multi-omics with respect to their positions on the MLS. Its purpose is to assign a high score to the multi-omics that have two properties at the same time: they are adjacent on the sequence and strictly connected on the pathway. The algorithm assigns a score equal to zero to the multi-omics that are adjacent on the sequence but unconnected to the pathway. In Fig. 5, the algorithm weights two adjacent multi-omics evaluating their positions (i.e. *j* and *j*+1 on the pattern *mov*_*i*_=[*e*_1_,*e*_2_,…,*e*_*j*_,*e*_*j*+1_,…*e*_*n*_]). A high score is assigned when the multi-omics *e*_*j*_ and *e*_*j*+1_ are directly connected on the pathway (green dotted lines) or with the shortest path (magenta dotted lines), otherwise a score of 0 is assigned. The multi-omic reciprocal adjacency is computed for all the adjacent couples *c*. The median value of the MORA weights is a summary index of the reciprocal influences between multi-omics and underlines the interplay among them on the MLS. Note that in multi-omic sequence the propagation of influences of an element becomes gradually weaker as the distance from its neighbouring elements increases: the propagation of influences is limited to the metabolic network average path length (APL) (see Additional file 1: Section S5 - Section *5.0.4*), decreasing its influence gradually and in an inversely proportional way with respect to the path length. If sequences and pathways do not present reciprocal influences (*RI*=0), the oscillating multi-omics lose their significance with respect to the pathways and vice-versa. In these cases, MLS interactions do not present a real biological meaning.

MORA algorithm takes as input the following parameters: *mov*^*i*^, which is the pattern of the i-th MLS, *G*^*i*^, which is its pathway and APL. Let us define *δ*=2 as the distance on the sequence from *e*_*j*_ to *e*_*j*+1_,∀*j*∈ [0,*n*−(*δ*−1)]. *δ* is fixed to 2 and identifies the adjacency of positions *j* and *j*+1 on the pattern (as it is shown in Fig. 5). Iteratively, each couple of (*e*_*j*_,*e*_*j*+1_) is associated with a couple of nodes in the pathway with the same index for simplicity: (*p*_*j*_,*p*_*j*+1_). Then, the algorithm will search in *G*^*i*^ the shortest paths with the end nodes *node*_*from*_=*p*_*j*_ and *node*_*to*_=*p*_*j*+1_. Also, we define *ψ*∈ [1, ⌈*APL*⌉] as the path distance between *p*_*j*_ and *p*_*j*+1_. For example, in Fig. 5, MORA finds a direct link between the pathway nodes (green dotted lines) and a path of length 2 (in magenta dotted lines).

We define the array of weights *infl* as an array of all zeros with the same length of *mov*^*i*^. When the algorithm finds the shortest paths on *G*^*i*^ from *node*_*from*_ to *node*_*to*_, it takes the positions *z* of the path nodes on the *mov*^*i*^ and in correspondence of these positions he gives a weight to the vector element *infl*_*z*_ (i.e the algorithm in Fig. 5a takes for *ψ*=2 the positions *z*={*j*,*j*+1,*h*}). The weights are computed with the following formula: $ w = \frac {1}{\psi _{y} -1} $, for the *y* -th shortest path found. These weights are summed up in the vector *infl* as follows: *infl*[*z*]=*infl*[*z*]+*w*. If there are *c* couples with *δ*=2 and *ψ*=2 then a perfect adjacency on *mov*_*i*_ and a direct edge on *G*_*i*_ are present, and then *w*=1. The weight *w* becomes progressively smaller if the distance *ψ* between the end nodes increases. The maximum distance between the end nodes is limited by the upper bound that is properly ⌈*APL*⌉. The supplementary Section S6-Figure 4 (Additional file 1) describes the whole procedure of the algorithm with a toy example and a related Figure. MORA is tested against several extreme structures proving its correctness. In particular, MORA is shown to be stable in case of cliques (supplementary Figure 4 (Additional file 1) part **(b)**), in *snaked* networks (Additional file 1: Figure S4) part **(c)** and on complete networks (not shown).

MORA is given as yardstick for validating and deciding if the measures that are taken in next Sections for oscillating multi-omic patterns and pathways are comparable and at what level of reliability.

Algorithm 1 presents a schematic description of the MORA methodology. The code is available at https://github.com/lodeguns/MORA.


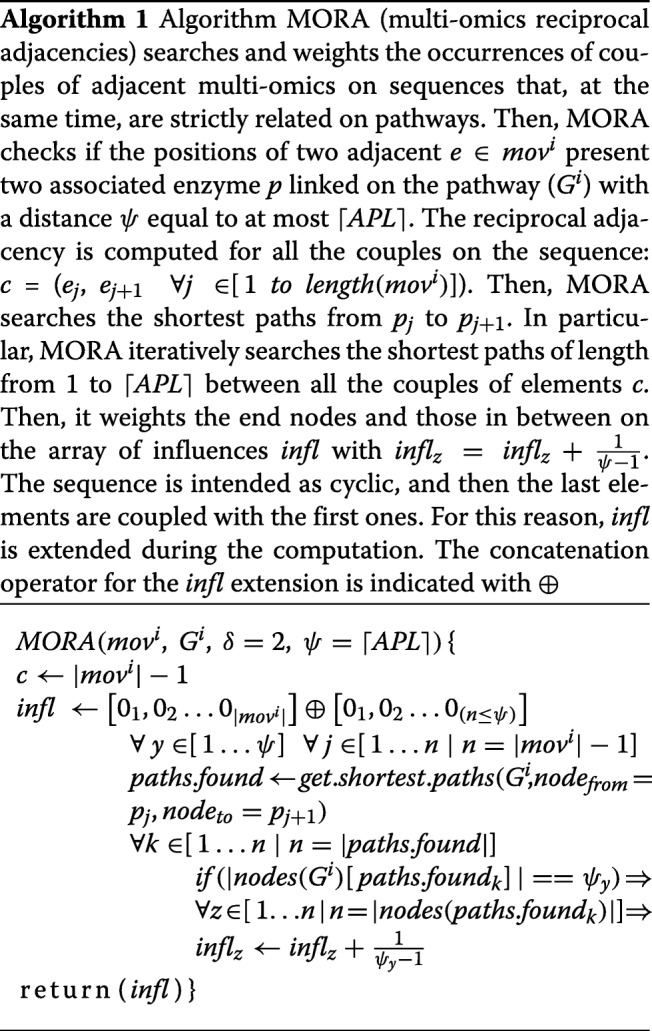


### Oscillating multi-omics on patterns

In this work, the multi-omics are discretized in a binary field as described in the previous “Binary discretization of multi-omics taking into account the global and the local effect” section, so that these values are classified into two classes (*N*=2). A multi-omic pattern presents an alternation if the two adjacent *e*_*j*_ and *e*_*j*+1_ in the pattern sequence are subtracted and ∣*e*_*j*_−*e*_*j*+1_∣=1. If the result of the subtraction is 0 then *e*_*j*_ and *e*_*j*+1_ are equal and there is not an alternation (i.e. Fig. 1d*(2)****a***_**1**_).

Furthermore, *l* is defined as the length of *mov* and *div*=*l*−1 is the number of divisors between the *e*_*j*_. For each pattern, the relative multi-omic pattern score *a*.*s* (Eq. 4) is the number of adjacent alternated couples of values divided by *N*:

4 $$ a.s = \sum_{j=1}^{div} \mid{e_{j} - e_{j+1}}\mid\cdot\frac{1}{N}  $$

We are interested in patterns of oscillating multi-omics with values close to the maximal score. We can obtain the alternation score of multi-omic patterns leveraging Eq. 4. A maximal score corresponds to a sequence of fully oscillating multi-omic values, for example *mov*_*s*_=0−1−0−1−...0−1 or alternatively *mov*_*s*_=1−0−1−0....1−0−1−0. Equation 4 returns the maximal score if and only if the pattern sequence presents fully oscillating multi-omics. The correctness of the last statement is proved in Theorem 1 (Additional file 1: Section S4 - Theorem 1). Fully oscillating sequences of different lengths, *l*_1_ and *l*_2_, present different scores depending on their length. Thus, it would be better to normalize the score dividing it by the number of divisors. Then, the absolute score of alternation is obtained, as in Eq. 5.

5 $$  w.s = \frac{\sum_{j=1}^{div} \mid{e_{j} - e_{j+1}}\mid\cdot\frac{1}{N}}{div}  $$

The maximal absolute score is proved to exist and it is unique for each pattern sequence with a specific length (Additional file 1: Section S4 - Theorem 2). Theorems 1 and 2 are important because they prove the correctness of how to compute the distance *d* of an observed oscillating multi-omic pattern *mov*_*obs*_ (i.e., *mov*_*obs*_:1−0−1−0−0−1−0−1−1−0) from the maximal absolute score (that can be considered as the ideal score). Thus, we have a powerful instrument to investigate how much the oscillations observed in *mov*_*obs*_ are found to be distant from the ideal (maximal) oscillations *mov*_*id*_. The distance *d* in Eq. 6 is the classical Manhattan distance:

6 $$ d = N \cdot (w.s({mov}_{id}) - w.s({mov}_{obs})) \geq 0  $$

*d* is a geometric distances only if *mov*_*obs*_ and *mov*_*id*_ have the same length. The similarity index *mov*_*obs*_ with respect to *mov*_*id*_ is computed using Eq. 7:

7 $$  \sigma_{obs} = 1 - d  $$

### Oscillating multi-omics on networks

In this Section, we illustrate the effect of oscillating multi-omics when projected on pathways. We consider the Park and Barabási [35] *dyadic model* on the pathways. In particular, a couple of proteins, linked in the pathway, presents a dyadic property if they both have the same multi-omic value (i.e., the couples of red-red nodes or blue-blue nodes in Fig. 1b*(1)*). Instead, they show an anti-dyadic property if they have oscillating multi-omics (couples of red-blue nodes). Following the model of Park and Barabási, it is possible to compute the expected value of an anti-dyadic effect on the couples of nodes that present an alternation. Let *n*_1_ be the number of pathway nodes with multi-omic values equal to 1, and *n*_0_ be the number of pathway nodes with multi-omic value equal to 0. In this way, the total number of nodes in the pathway is *N*=*n*_1_+*n*_0_. The number of links between nodes that show the anti-dyadic property are described by the variables *m*_10_ and *m*_01_. The number of links between nodes that do not show an alternation (dyadic property) are represented by the variables *m*_11_ and *m*_00_. Therefore, the total number of edges for a pathway is *M*=*m*_10_+*m*_01_+*m*_11_+*m*_00_. Note that the maximal possible number of edges in a directed network is equal to *N*(*N*−1). The network density *δ* [36] of a directed graph is described in the following Eq. 8:

8 $$  \delta_{p} = \frac{M}{N(N-1)}  $$

The random variables $X_{m_{10}}, X_{m_{01}}$, $X_{m_{11}}$, $X_{m_{00}}$ (where $X_{m_{10}} = X_{m_{01}}$) describe the events of oscillations or non-oscillations in a network. The expected value of observing an alternation (anti-dyadic property) on two nodes given by Eq. 9 is the following:

9 $$  {}E\left[X_{m_{10}}\right] = E\left[X_{m_{01}}\right] = \binom{n_{0}}{1}\binom{n_{1}}{1} \frac{M}{N(N-1)} = n1 \cdot n_{0} \cdot \delta_{p}  $$

On the other hand, the expected value of not observing an alternation (dyadic property) is defined by Eqs. 10 and 11:

10 $$  E\left[X_{m_{00}}\right] = \binom{n_{0}}{2}\delta_{p} = \frac{n_{0}(n_{0} -1)}{2} \delta_{p}  $$

11 $$  E\left[X_{m_{11}}\right] = \binom{n_{1}}{2}\delta_{p} = \frac{n_{1}(n_{1} -1)}{2} \delta_{p}  $$

The last three Equations describe the link of every pair of nodes with a given probability [37, 38] as described by the Gilbert’s model for random networks. Therefore, if the counts of *m*_10_, *m*_01_, *m*_11_ and *m*_00_ deviate from the expected values described above, then it is possible that the multi-omics on the pathways are disposed in a structured manner, differently from Gilbert’s model. The ratio between the observed alternation and its expected value is a direct measure of deviation (a.k.a. magnitude) of the observed pathway from a random configuration. In particular, the pathways present oscillating multi-omics with a magnitude $\widehat {m_{10}}$ given by Eq. 12 :

12 $$ \widehat{m_{10}} = \frac{m_{10}}{E\left[X_{m_{10}}\right]}  $$

Following the properties of the network structure, if the nodes present a dyadic effect, their magnitudes $\widehat {m_{11}}$ and $\widehat {m_{00}}$ are given by Eqs. 13 and 14:

13 $$ \widehat{m_{11}} = \frac{m_{11}}{E\left[X_{m_{11}}\right]}  $$

14 $$ \widehat{m_{00}} = \frac{m_{00}}{E\left[X_{m_{00}}\right]}.  $$

If $\widehat {m_{01}}$ is greater than 1, it indicates that multi-omic nodes are oscillating more than expected by a random configuration. Multi-omics are not oscillating in the same way when $\widehat {m_{00}} > 1$ or $\widehat {m_{11}} > 1$. Due to the configuration of the pathways, it is possible that the anti-dyadic magnitude (see Eq. 12) and both the dyadic magnitudes (see Eqs. 13, 14) present values greater than 1; therefore, a network is *mainly oscillating* if $\widehat {m_{10}} > \widehat {m_{11}}$ and $\widehat {m_{10}} > \widehat {m_{00}}$.

The *average dyadic-effect*$\widehat {m_{0011}}$ is defined as the average between the magnitudes $\widehat {m_{11}}$ and $\widehat {m_{00}}$.

### The whole procedure performance

Given an unweighted graph *G*(*N*,*E*), with *N* the set of pathway nodes and *E* the set of edges/reactions, MORA searches the reciprocal influences with a polynomial complexity on *N*. Additionally, MORA is coupled with the measurement of oscillations making the whole procedure exponential. The function *get.shortest.paths* is a modified function that implements a breadth-first search (BFS) structure [26] and takes a track of all the shortest paths between two end nodes. The worst case time complexity for this search algorithm is of **O**(|*N*|+|*E*|), but 0≤|*E*|≤|*N*|, thus, the complexity could be quadratic on N: *O*(|*N*|^2^). The latter is called for each adjacent couple (*N*−1) of multi-omics along the sequence plus the one taken considering the couples between the first and the last elements (*O*(|*N*|(|*N*|+|*E*|)) [39]. Moreover, the computation of the dyadic/anti-dyadic effect requires an exhaustive enumeration of all the possible configurations of the *n*_1_ nodes on the whole node set N: $\binom {N}{n_{1}} = O\left (N^{n_{1}}\right)$. The latter turns out to be exponential in time but it is possible to apply an approximation by analyzing the boundary configurations of a phase diagram [35]. Unfortunately, in this case, due to the high specificity of enzyme-substrate reactions, with the latter approximation a large part of the biological information is lost. The computation of the oscillating multi-omics on sequences require a linear time with respect the number of nodes (*O*(|*N*|)). As consequence, the whole procedure is of **f**$\left (\mathrm {N}, n_{1}\right) = O\left (N^{n_{1}}\right) + O\left (|N|^{3}\right)) + {O}(|N|)$.

### Data sources and multi-omics data integration

In the Additional file 1: Section S3 the data sources and the differences between static and dynamic omic values are described. Static omic values, as CAI, are those not responsive to antibiotics. Dynamic omic values are those sensitive to treatments, as, for example, mRNA amount, protein abundance and its variation. In particular, for what concerns the transcriptomic layer, the microarray compendia are extracted with relevant between-studies reliabilities [40] as described in the Additional file 1: Section S5 *pp.5.0.2*. Furthermore, in the proteomic layer, a random effect model is designed with affordable predictors with the aim of obtaining a noise-robust protein variation *pv* as a fundamental dynamic omic (See [41] and Additional file 1: Section S5 *pp.5.0.3* for more details on the model). For what concern the metabolomic layer, a novel protein-centric network of reactions is obtained by integrating two sources [42, 43] (Additional file 1: Section S5 *pp.5.0.4*). Finally, in the genomic layer, relevant information comes from the codon usage extraction as described in the Additional file 1: Section S5 *pp.5.0.1*.

## Results

The experiments were performed on 66 pathways in standard conditions and taking into account the average effect of 69 antibiotic perturbations.

The sequence patterns and network motifs are studied on 66 multi-layered structures four times. In fact, four different experimental set-ups were compared on the same dataset: (i) MLS without modifications; (ii) MLS with operon compression; (iii) MLS with path extension; (iv) MLS with operon compression followed by path extension. (see violet block in Fig. 2)

The MORA algorithm evaluated the reciprocal influences *RI* on the 66 x4 MLS (see white block in Fig. 2). Once the associations between the recurring patterns and network motifs were evaluated, the oscillating multi-omics were computed with the following scores: (i) the similarity between observed oscillating multi-omic patterns and the ideal patterns (Eq. 7); (ii) the pathway dyadic/anti-dyadic effect magnitudes, as illustrated in Eqs. 12, 13, 14 (see also green blocks in Fig. 2).

For a detailed description of the experimental parameters see in the Additional file 2: Tables S1 and S2. Row names are labelled with their unique E.*coli* KEGG identifier codes (*eco*:*pathNNNNN*).

### A small case study

Multi-omic oscillations of E.*coli* Glycolysis are shown in the Additional file 1: Figure S5 Section S7. In this case, in standard conditions, the similarity computed as in Eq. 7 is =0.6 (red line) and the $\widehat {m_{01}}$ is =1.9, while the magnitude of the dyadic effect $\widehat {m_{0011}}$ is =1.5. These values could change due to the perturbations, as shown in Additional file 1: Figure S5 part **(b)**. In this small case study, a single pathway *alternation* analysis with the effect of path extensions is shown in Additional file 1: Figure S6 highlighting that with or without path extensions, the oscillations on motifs are preserved.

#### Multi-omic oscillation features are present in standard conditions and show a multi-scale behavior

For the 66 pathways and supra-pathways extracted from KEGG the multi-omic oscillations in standard conditions through their associated 66 MLSs and relative modifications are studied.

##### Network Motifs:

**Fig. 6:**
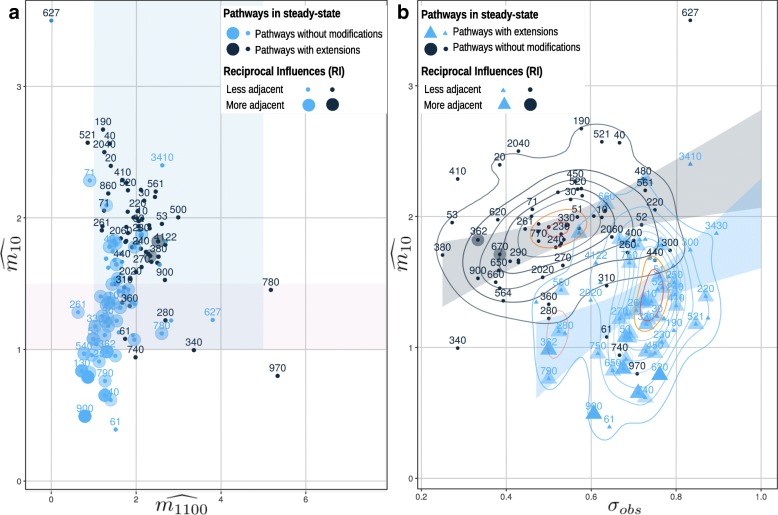
The anti-dyadic effect magnitudes (y-axis) and the dyadic effect magnitudes (x-axis) of 66 pathways are shown in Figure **a**. The pink rectangle underlines the area where the pathways present an anti-dyadic effect $\widehat {m_{01}}\ge 1$, instead the blue rectangle individuates the area where the average dyadic effect is $\widehat {m_{1100}} \ge 1$. The pathways with path extensions are shown with blue dots while black dots depict the same pathways without deviations. The number on the dots is the KEGG pathway identifier without its suffix *path:eco*. In Figure **b** the anti-dyadic effect is shown on the y-axis $\widehat {m_{01}}$ and the pattern similarity *σ*_*obs*_ to an ideal oscillating multi-omic pattern on the x-axis. Black dots describe pathways without extensions, and triangles depict those with extensions. The black and blue curves correspond to the two-dimensional kernel density estimation both for the dots and for the triangles. The plot is clearly separable with a binary classifier, individuating principally two bands (the black and the blue ones). Both the plots show that pathways without extensions have a median reciprocal influence =1±0.27 per node. Instead, pathways with extensions present a median reciprocal influences of =2±0.62 per node. Pathways with extensions present present better MORA reciprocal influences than pathways without extensions. The pathway is presented with a big shape on the plot if the *RI* is > 1.5 (more adjacent). Opposite, pathways with *RI* ≤ 1.5 are classified as less adjacent

It is clearly shown (Fig. 6a) that most of the pathways without path extensions (black dots) presents a relevant anti-dyadic effect (median $\widehat {m_{01}} = 1.87, sd= \pm 0.57$) even if it is also present a relevant but more variable dyadic effect (median 2.03,*sd*=±0.81). In the pathways with path extensions (blue dots) the anti-dyadic effect is decreased with a median 1.35,*sd*=±0.48; contextually, also the average dyadic effect is decreased (median 1.45,*sd*=±0.57). In some cases, path extensions, more than other MLS modifications, could increase the oscillations. In some other cases MLS modifications introduce new proteins as nodes and new edges as reactions that could decrease the oscillations; it depends on the network topologies. In this case, in the most part of the pathways, the anti-dyadic effect magnitude is decreased even if it remains relevant. In standard conditions, pathways without path extensions present only 18/66 combined MLSs with a *σ*_*obs*_>0.7 and $\widehat {m_{01}} >1$.

##### Sequence Patterns:

In Fig. 6b it is shown how combined and competitor structures interact. Furthermore, pathways with path extensions (blue triangles) are more oscillating on patterns (median *σ*_*obs*_=0.73,*sd*=0.10) with respect to those without extensions (black dots) (median *σ*_*obs*_=0.53,*sd*=0.14). On one hand, pathways with path extensions present a high anti-dyadic effect, on the other hand, they decrement the *σ*_*obs*_, moving the density center to a value nearer to 0.5. This means that pathways with path extensions seem to be more in combination than pathways without extensions. Multi-omic pattern operon compression returns similarity scores which are a little higher (median *σ*_*obs*_=0.54,*sd*=0.15) than in unmodified MLSs. Modifying MLSs, coupling operon compression and path extension, leads to lower oscillations in patterns (median *σ*_*obs*_=0.70,*sd*=0.12). The presence of operons on the patterns does not cause always the same effect: due to the compression, in some pathways, for example Glycolysis (KEGG identifier path:eco00010) *σ*_*obs*_ changes from 0.62 to 0.66, while, in the Citrate cycle (TCA cycle) path:eco00020, *σ*_*obs*_ changes from 0.38 to 0.62. In other cases, *σ*_*obs*_ decreases its value from 0.64 to 0.38, as, for example, in the Lysine degradation (KEGG identifier path:eco00310). After all, oscillation is still present for the most part of the pathways without the MLS modifications. In this way, multi-omic oscillations allow to uncover similarities between the network structures, which can reveal the existence of generic organization principles. A comparison of MLSs with and without modifications reveals a multi-scale presence of oscillations in sequences and networks of different dimensions but with a widespread tendency to homeostasis.

#### Multi-omic oscillations are related to MLS reciprocal influences showing a particular interplay between the sequence gene order and the pathway structure

It is possible to assess that MLSs are related to multi-omic oscillations underlining the interplay between the gene order and the particular schema of reactions. In both the plots of Fig. 6, pathways without extensions maintain a relevant reciprocal influence (median 1,*sd*=±0.27), increasing their value for all the pathways with extensions (median 2,*sd*=±0.63). In both the cases, the reciprocal influences are not near to 0, thus associating the anti-dyadic motifs to the neighboring multi-omics along the patterns. In this way, it is also proved that the network plasticity [19] does not depends only on a particular circuit of reactions but could be investigated through the extent of the MORA reciprocal influences between the sequence gene order and the network structures. From an evolutionary point of view, the sequence gene order and the pathway structures are conserved along the organisms [44, 45]. In future works, it could be interesting to investigate the interplay of gene order and network structures on several species taking as a measure of the evolutionary pressure the comparisons between MORA reciprocal influences.

#### Multi-omic oscillation features change in configuration due to perturbations and reveal different regulatory behavior

**Fig. 7:**
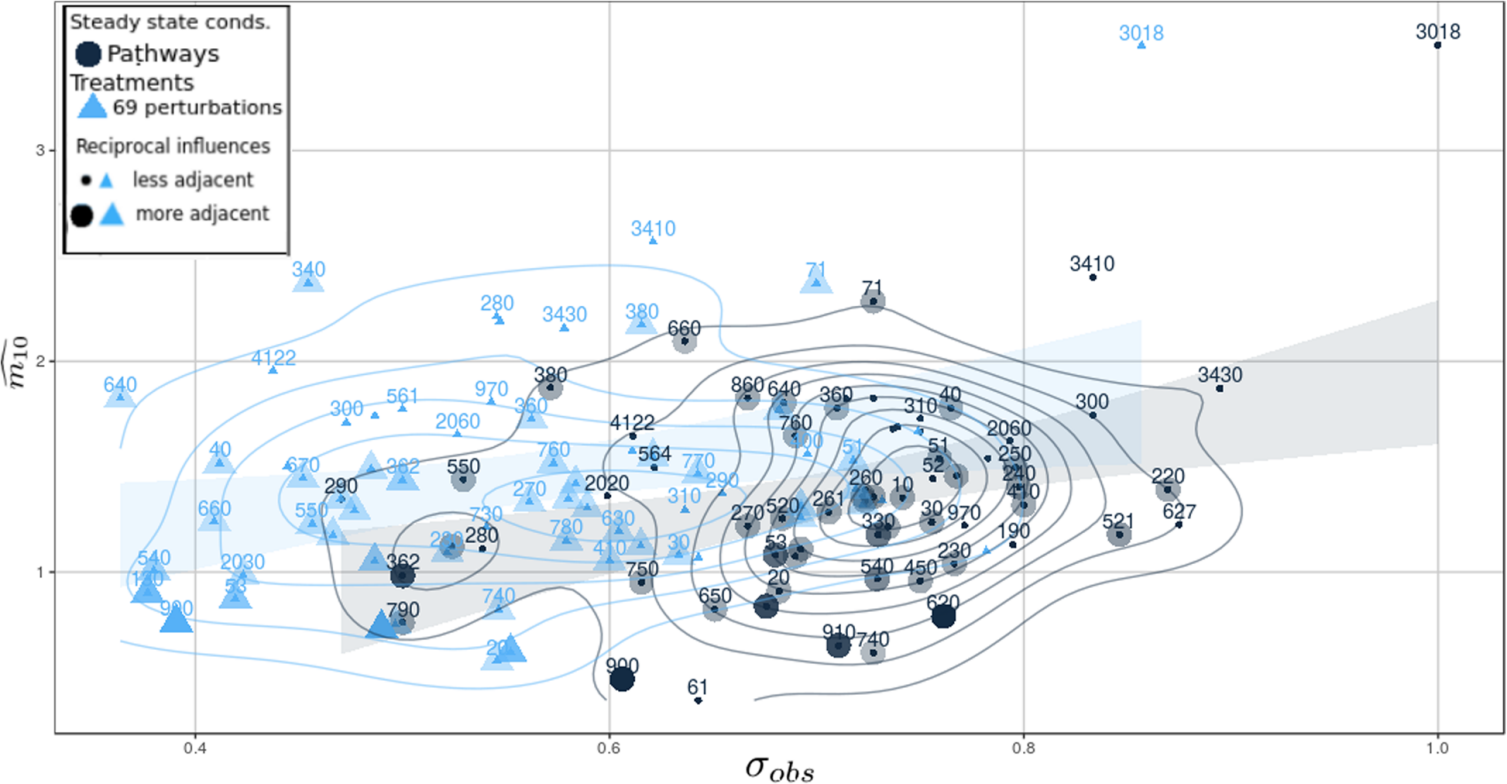
The anti-dyadic effect magnitudes $\widehat {m_{01}}$ (y-axis) and the similarity score magnitudes *σ*_*obs*_ (x-axis) of 66 pathways are shown in Figure. The 66 pathways with extensions subject to the average effect of 69 treatments are shown with the blue triangles. The same pathways in steady state conditions are represented with black dots. The pathway is presented with a big shape on the plot if the *RI* is > 1.5 (more adjacent). Opposite, pathways with *RI* ≤ 1.5 are classified as less adjacent

**Fig. 8:**
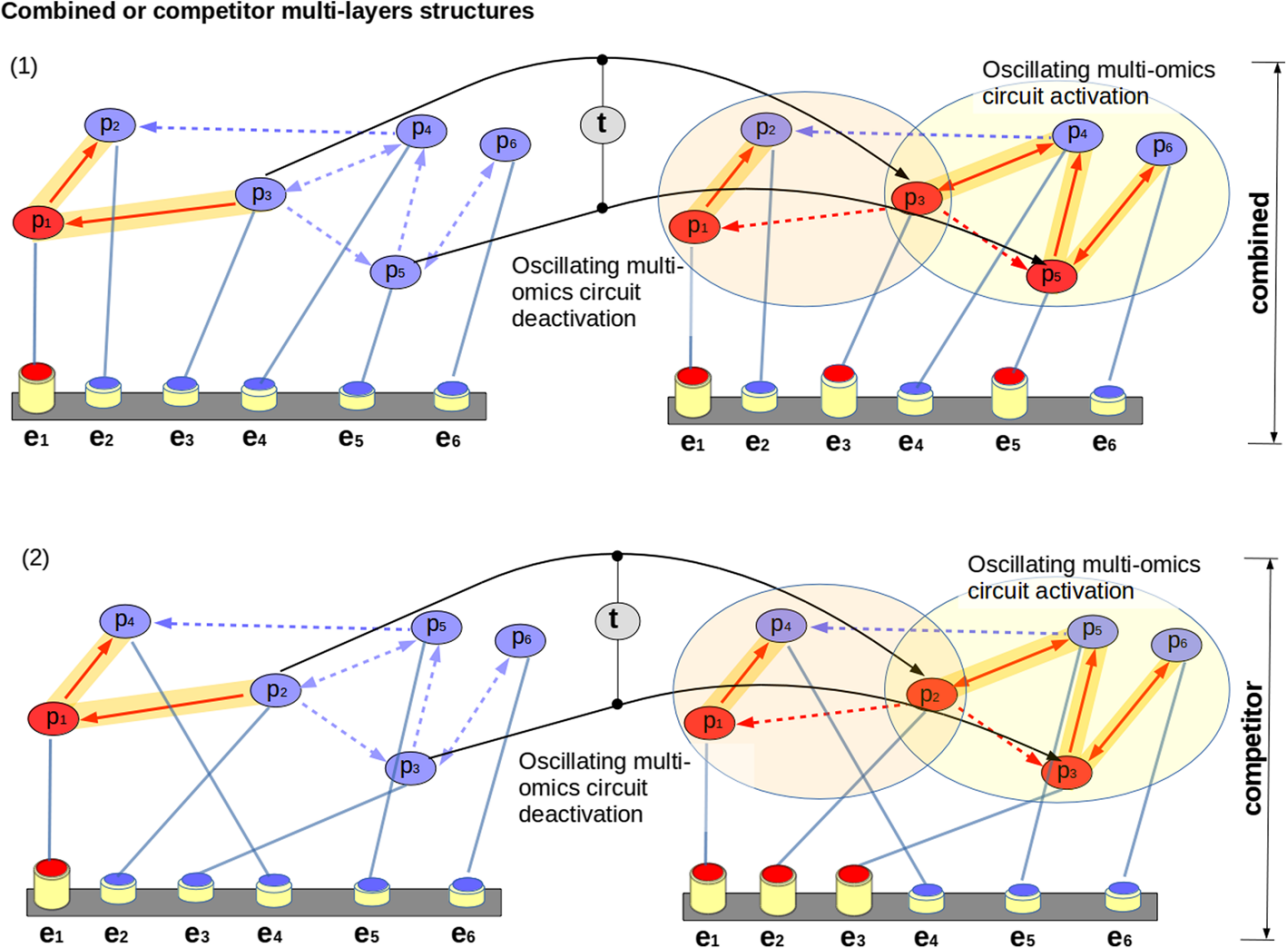
An MLS with high reciprocal influences (*RI*) but with a low number of oscillating multi-omics is shown in *(1)*. On this MLS, due to the effect of the treatments *t*, an oscillating multi-omic circuit is activated (orange links in the yellow circle) and is deactivated another one. The MLS become combined due to the effect of *t* because both the pattern and the pathway show oscillating multi-omics at the same time. Opposite, in *(2)*, the effect of treatments activate and deactivate the same oscillating multi-omic circuits, but, due to the changed pattern elements order, only the pathway shows oscillating multi-omics, instead the pattern shows a low number of oscillations. In this case, the structures are defined competitor. The change of only two multi-omic values (*p*_3_ and *p*_5_) on the overall pathway and on the pattern (*e*_3_ and *e*_5_) affect the whole recurring multi-omic pattern and its MLS

We measured the multi-omic oscillations on the 66 MLSs perturbed by considering the average effect of 69 antibiotic perturbations. A strong response from the MLSs, as shown in Fig. 7, is obtained when pathways with extensions in standard conditions (black dots) are compared against the average effect of 69 treatments (blue triangles). The treatment effects strongly lower the patterns oscillation score moving the median from *σ*_*obs*_=0.72,*sd*=0.10 to *σ*_*obs*_=0.57,*sd*=0.11. On the other hand, on the networks, they led the median values of $\widehat {m_{01}}$ from 1.36 to 1.41, with an increase in variability from *sd*=0.50, to 0.87. These values show that the organism, in response to the treatments, activates other oscillating circuits on the same pathway, silencing some others. For example, in Fig. 7, base excision repair (KEGG pathway id eco03410 with extension) reasonably increases its anti-dyadic effect of more than 0.5. Mismatch repair (KEGG pathway eco03430) also increases its anti-dyadic effect magnitude, and in this case, its oscillating similarity on the pattern is lowered. The same is for the Flagellar assembly pathway (KEGG pathway eco02040) that, due to the label overlapping not shown on the plot, in standard conditions, is near to the black node 360 while in the treatments it is behind the blue triangle 280, always in Fig. 7. The structures (sequence and pathway) that form the MLS are defined *in combination* if oscillating multi-omics are present at the same time on the patterns and on the network motifs. Instead, when oscillating multi-omics in one structure are found on patterns and not on pathways or vice-versa, then these structures are defined *in competition*. In our case, it is observed that the *combined structures* in standard conditions are 47/66 considering pathways with extension, those with operon compression are 14/66, while those with operon compression and extension are 43/66. The average effect of the 69 treatments causes only 25/66 MLSs in combination. Similar results are obtained considering MLSs with operon compression and with operon compression and path extension. Some structures that are combined in steady state conditions due to the perturbations become competitors and vice-versa. Configuration changes imply the activation or the deactivation of oscillating multi-omic circuits on the pathways (as shown in Fig. 8) highlighting the presence of different and unbalanced regulatory functions.

## Discussion

The proposed methodology with regard to performances could be comparable with well-known mining methodologies of sequence patterns [46] and dyad motifs [35, 47]. From the biological point of view, it is based on an extensive perturbation multi-omic analysis related to network and sequence structures. In this field, recent studies are focused on frequency patterns, that, conditioned by biochemical oscillators, are activated or deactivated on regulatory network circuits. In particular, it is proven, that different types of regulatory functions appear to be related to particular network structures to such an extent that different biochemical oscillators are associated with specific structures [48]. Furthermore, some other studies are focused on bacterial network motifs and sequences which deepen the topological features on complex structures [49]. As a consequence, our methodology is focused on multi-omic patterns and motifs by putting them in a biological and structural relation. In this way, it is possible to leverage the topological interplay between networks and sequences for understanding the effect of perturbations and the role of regulatory functions. Consequently, discovered patterns and motifs are considered part of the same multi-layered structure (MLS) and they come from quite easily data integration processes useful to compare dynamic omic sources and perturbations (protein variation, mRNA amounts, etc.) with static omic sources (codon usage, gene order, pathways). Therefore, it is analyzed the extent of oscillating multi-omics and their reciprocal influences investigating whether sequence patterns and network motifs are combined or competitor. In this setting, we have shown how perturbations alter the MLS dynamically. These changes were proved to trigger the activation and deactivation of oscillating multi-omic circuits with major implications in the metabolic response to antibiotics. Moreover, unknown structural features are revealed, e.g. it is shown that the gene order and the bacterial reaction circuits reveal a strong interplay in combined structures. MLSs were studied when subject to modifications (operon compression and path extensions) considering multi-scale multi-omic metabolic networks. When the motifs present a lack of oscillating multi-omics, then through the path extensions we have seen how the metabolic network affects the recurring patterns more than the operon compression. In this setting, some patterns show high scores while others not. The reason is that, in some cases, the network helps the recurring pattern to maintain a structural oscillation, while, in other cases, the network influences negatively the oscillations. In this way, the variable multi-omic quantities could be analyzed in order to discover unknown regulation mechanisms between the different omic-layers. Impressively, competitor MLSs, after a perturbation, could become combined showing a sort of synchronization used to realize a catabolic or an anabolic process in an optimized manner. Furthermore, the latter observations coupled with the discretization of multi-omics depict the complexity of metabolic processes and their response to antibiotics unveiling the rules behind the metabolic network robustness and plasticity [19]. After all, MORA reciprocal influences between motifs and patterns are obtained in a reasonable time (see 2) on an almost complete dataset. In fact, the whole protein-centric network is of 1.644 vertices and 369.863 edges and, in our knowledge, it does not exist a larger network (in terms of vertices) on which to project more multi-omics. The selected experiments come from two compendia specifically suited for antibiotic treatments (see Paragraph Data sources and multi-omics data integration). In order to make results comparable, it is possible to extend the whole procedure to other experiments considering perturbations of the same typology. Unfortunately, the complete proposed methodology is exponential (see Paragraph The whole procedure performance) because of the measurement of oscillating multi-omics on networks (see Paragraph Oscillating multi-omics on networks). It is possible to achieve ad hoc algorithmic improvements reducing the pathway redundancy [50] or saving and reusing the computations done on overlapped pathway sub-circuits/sub-graphs in specialized data-structures. After all, functional plasticity, homeostasis, redundancy, and promiscuity conserved biological aspects of metabolic networks and biological processes which makes it possible to survive to external perturbations [51]. Therefore, it is a better choice to not remove redundancy preserving the biological mining of the research. For the same thing it is recommended to pay attention in the network edge pruning due to the promiscuity of small metabolites (i.e *H*_2_*O*,*O*_2_,*AMP*, etc …) [25, 52]. Oscillating multi-omic patterns associated with dynamic rates of substrate/compound transformations might lead to predicting some specific biochemical processes also in presence of missing data, by using inferential methods based on the profiling of reaction velocities and their dynamics. Future improvements might lead us to study oscillations with *N*>2 classes. In literature there are several methodologies to define *N* as a discrete space of features [53]. We suggest to apply a consensus criterium in order to project multi-omics on a discrete space with *N*>2 because there is not an a priori best methodology. For example, we adopted the Sturge’s formula in one of our precedent works [12]. Once the number of discretisation levels *N* is decided, the procedure to obtain *σ*_*obs*_ is very similar to the one described in the paragraph Oscillating multi-omics on patterns. Note that if we have a set of discretization levels *Σ*={0,1,2,..*N*}, then each discretisation level contributes to the pattern oscillation by its fraction 1/|*Σ*|. The ideal *σ*_*obs*_ is equal to $\frac {(max(\Sigma)-0) * div}{max(\Sigma)*div} = 1$. It follows that the ideal oscillation with *N*>2 assumes the form of a series of min-max discretisation levels: *max*(*Σ*)−*min*(*Σ*)−*max*(*Σ*)…*min*(*Σ*)−*max*(*Σ*). More the adjacent values are nearer to *max*(*Σ*) or *min*(*Σ*) the less we have oscillating multi-omics. The multi-class dyadic effect follows the same rules presented above where the maximal oscillation is equal to *N* and the others are ∈ [0,*N*].

## Conclusion

In this paper a multi-omic integration based methodology is introduced to analyse bacterial oscillating multi-omics on sequence patterns and network motifs. The subject of our analysis is E.*coli*. The lack of methodologies for multi-omics integration decreases the chance to detect emerging motifs and patterns across sequences, networks or pathways. The current need of analytics to compare and test metabolic models, the accurate design of a new pathway or the redesign of an existing metabolic pathway or the experimental validation of an in silico metabolic model using a nearby species require the information of how multi-omics data build up multi-scale phenotypes. The high level comparison is based on novel algorithms which take the low level approach at pathway level framework and generate multi-scale multi-omic metabolic networks. The goal is to find relations between oscillating multi-omic patterns on sequences and oscillating motifs on metabolic networks. Recurring oscillating multi-omic patterns are discovered to be related to multi-omic metabolic network motifs. This last novel feature can be related to the highly conserved gene order and to the highly specificity of enzymatic reactions and their network topology. The discovered motifs can be not only useful in the study of bacterial phenotypic responses but also in applications of metabolic engineering and optimizations. Then, this work can contribute to the study and to the creation of new interesting metabolic circuits based on binary multi-omic network motifs and their recurring patterns. Our framework is implemented in a software written in R which provides effective and friendly means to design intervention scenarios in the perspective of the synthetic biology.

## Additional files

Additional file 1 This file describes all the technical details and procedures used for the multi-omic data-integration. In addition, some Sections show some math proofs for what is concerning the oscillating multi-omics, a toy example for the multi-omic relational adjacencies and some concrete examples of pathway and patterns. (PDF 767 kb)

Additional file 2 It is a PDF document which contains 66 measures of dyadic/anti-dyadic effects, oscillation similarity scores, and reciprocal influences computed with MORA. Data are reported in standard conditions and considering the average effect of 69 treatments. (PDF 108 kb)

## Abbreviations

Alphabetical list of abbreviations: APL: Average path length
CAI: Codon adaptation index
FB: Francesco Bardozzo
MLS: Multi-layered structures
MORA: Multi-omic reciprocal adjacencies
PA: Protein abundance
PL: Pietro Lió
PV: Protein variation
RT: Roberto Tagliaferri
