# Supplementary material for: A study on multi-omic oscillations in Escherichia coli metabolic networks
Source: BMC Bioinformatics. 2018 Jul 9;19(Suppl 7):194. doi: 10.1186/s12859-018-2175-5 (PMC6069781; doi:10.1186/s12859-018-2175-5)
Supplement: Supplementary file 1 — This file describes all the technical details and procedures used for the multi-omic data-integration. In addition, some Sections show some math proofs for what is concerning the oscillating multi-omics, a toy example for the multi-omic relational adjacencies and some concrete examples of pathway and patterns. (PDF 767 kb) [file 12859_2018_2175_MOESM1_ESM.pdf]

The study of multi-omic oscillations in Escherichia coli metabolic networks

Francesco Bardozzo
NeuRoNe Lab, DISA-MIS
Via Giovanni Paolo II 132
Salerno IT

Pietro Lió
Computer Laboratory
15 JJ Thomson Ave
Cambridge, UK

Roberto Tagliaferri
NeuRoNe Lab, DISA-MIS
Via Giovanni Paolo II 132
Salerno IT

November 8, 2017

1 S1 - Criteria for deciding the number of $mRNAs/cell$

The number of $mRNAs/cell$ is constantly object of study because of several experimental settings. Bartolomaus et all. provide two $mRNAs/cell$ quantities in different experimental settings: upon osmotic stress in the minimum medium, the number of mRNAs decreases from $\approx$ 2400 mRNA $copies/cell$ to $\approx$ 1600 mRNA $copies/cell$ (this is our lower bound). Instead, upon an heat stress in nutritionally rich medium, it is recorded a reduction of $copies/cell$ from $\approx$ 7800 mRNAs to $\approx$ 7200 mRNAs [1] (this is our upper bound). Steady state conditions minus treatment conditions on average lead to a value between $\approx$ 2000 $mRNAs/cell$ and $\approx$ 7500 $mRNAs/cell$. Furthermore, E.*coli* under exponential growth at medium growth rate is known to contain about 3000 mRNA $copies/cell$ [2]. Thus, considering the different growth conditions, our scaling coefficient for the normalisation is considered as a fraction

of $\approx$ 3800 mRNA *copies/number* on the summatory of *totalRNA* for each microarray replicate. Note that this scaling factor does not change the mRNAs fold change and it is simply a change of measure units.

2 S2 - Criteria for the trasformation of protein abundance *ppm* in *proteins/cell*

Protein abundance furnished in *ppm* is transformed in $\frac{molecules}{cell}$. The first step is to compute the scaling factor to normalize the data. Then it is computed the molecular weight *gram/mole* for each protein: it is accomplished extracting the molecular weight of each amino acid, and then applying equation 1 according to the involved chemical quantities :

$$ppm_i * \frac{\frac{gram}{cell} \cdot \frac{molecules}{mole}}{\sum_j^n ppm_j \cdot \frac{gram}{mole}_j} = \frac{molecules}{cell} \tag{1}$$

In this case, at the numerator the total mass of proteins estimated per cell is of $2.35e6$ grammes per cell [2] and the constant implied in the conversion of molecules per mole is the number of Avogadro. The denominator of the equation represents the summation of the expressed protein abundances *grammes/moleperppm*. Then the scaled protein abundance is obtained in *molecules/cell* via the multiplication of the scaling factor times the i-th value of protein abundance expressed in ppm (ppm_i).

3 S3 - Static and dynamic multi-omics

We can separate omic sources in two category: the first one represent the data that could be intended as variable because are changing due to the effect of treatments, the second one represent the data that are static because does not change during perturbations. In all the cases omic data subject to perturbations (for example protein abundance or mRNA expression levels) and static omic data (like codon usage) maintain the same schema and the same identifier over all the sources. The names of the proteins and the structure of the pathways are extracted from the KEGG REST API [3] and EcoCyc[4]. The protein pathways of KEGG [5] can be used for the extraction and association of other types of information extracted from NCBI[6] and EcoCyc and

vice-versa. From Ecocyc are extracted the information about the operons and protein complexes, co-regulations, enzymatic functions and their direction of reaction: the latter will be of central importance when reconstructing a *protein centric* metabolic network. The NCBI information outlines genes and their positions on the DNA double strand; moreover, they give us information about the direction, 5'-3' or 3'-5', of these genes in the double strand that are presented as + and − in my multi-omic space. From this source it is extracted also the position of the genes on the double strand which is an intrinsic information fundamental for the definition of the multi-layer structure. For each gene name are downloaded the DNA sequences and it is computed the codon usage. We have described till now static omic sources. Instead, variable omic sources are extracted from NCBI Gene Expression Omnibus (GEO) [7] and protein abundance database PaxDB [8]. In particular, we have used a web-service called GEO2R, that in turn is based on GEOquery [9] and limma [10]. GEO2R is a very useful web-service through which it is possible to generate R [11] code for obtaining information about microarray experiments and gene expression profiles with a user-friendly web interface. From this service it is possible to obtain mRNA amounts in steady state conditions (controls) and after perturbations (treatments). Instead from PaxDb it is extracted the protein abundance in standard conditions. Leveraging the services of NCBI are unified the names that on the selected microarray are obsolete, thus avoiding the lack of collinear schema information. Coupling informations about mRNA controls and protein abundance it is inferred a protein variation (*pv*) as it is described in the paper.

4 S4 - Correctness of the relative and absolute score for oscillating multi-omics on sequences

4.1 Theorem 1

For each *mov* the relative multi-omic sequence score (equation 2) is just the number of adjacent couple of values divided by N:

$$a.s = \sum_{j=1}^{div} \mid mov_j - mov_{j+1} \mid \cdot \frac{1}{N} \tag{2}$$

Th 1: The maximal score from the equation 2 is obtained if and only if on a multi-omic pattern there are fully oscillating multi-omics.

Proof: The maximal score corresponds exactly to the expected value of a random variable X (see equation 3) defined as follows: each of the events $\mid e_j - e_{j+1} \mid$ can fall in 0 or 1 showing or not oscillating multi-omcis; therefore, this event is represented by a binary random variable X_i for all the $0 \leq i \leq div$ which represent the chances of seeing an oscillation of multi-omic values.

$$X_i = \begin{cases} 1 & if \mid e_i - e_{i+1} \mid == 1 \\ 0 & otherwise \end{cases} \tag{3}$$

Then, for each X_i, the expected number $E[X_i]$ of occurrences of an oscillation is equal to $E[X_i] = 1 \cdot (\frac{1}{N}) \; + \; 0 \cdot (1 - \frac{1}{N}) = \frac{1}{N}$, and it is similar to take into consideration a bound to the load balancing problem [12]. The expected number $E[X] = E[\sum X_i] \quad \forall\ 0 \leq i \leq div$ of seeing a fixed anti-dyadic effect on N classes for the div independent chances is a *balls and bins* problem where it is considered the max loading of a fixed machine [13] and in equation 4 it follows by leveraging the property of linearity for the i.i.d. X_i:

$$E[X] = E[X_0] + .. + E[X_i] + ... + E[X_{div}] = \frac{div}{N} \tag{4}$$

The correctness of this result is not difficult to prove. It is given for induction on the expected value $E[X]$. The base of the induction is for $i = div$ then for $E[X_{div}] = \frac{1}{N}$ we arrive to $E[0] = 0$. For hypothesis a single oscillation is founded with a chance of $\frac{1}{N}$ that corresponds to the chance of a ball to fall in a fixed bin. For our random variable of Equation 3 there are two outcomes: either a ball falls into the fixed bin and X_i adds 1 for the property of linearity of the expected value, or it adds 0. Therefore, we have:

$$E[X_{div}] = \frac{1}{N}(1 + E[X_{div-1}]) + \frac{N-1}{N}(0 + E[X_{div-1}]) \tag{5}$$

resembling equation 5 it is proved that $E[X_{div}] = \frac{1}{N} + E[X_{div-1}]$ and resolving this one for each i corresponds to obtain equation 4. In conclusion, the score of equation 2 in the case that all the observed multi-omic values in a sequence are oscillating (maximal score) it is equal to the expected value of X defined by equation 4.

4.2 Theorem 2

The absolute score 6 is obtained dividing the relative score (equation 2) by the number of divisors thus showing an absolute score oscillation measurment, as written in equation 6.

$$w.s = \frac{\sum_{j=1}^{div} \mid e_j - e_{j+1} \mid \cdot \frac{1}{N}}{div} \tag{6}$$

Th 2: The maximal absolute score on equation 6 is just the expected value of X_i and it is referred to the expectation of seeing an ideal oscillation where each 1 follows a 0 or vice-versa (i.e. $mov_{id} : 1-0-1-0-1-0-1-0-1-0$).

Prof : In the following equation 7, where $l - 1 = div$ it is proved the relation with the maximal absolute score and the expected value of X_i:

$$\frac{l - 1 \cdot \frac{1}{N}}{div} = E[X_i] = \frac{1}{N} \tag{7}$$

5 S5 - Multi-omic layers integration

5.0.1 Genomic layer: the role of codon usage

One of the most relevant omics considered is the codon usage. The latter is widely proved being a fundamental component showing relevant patterns on the genome [14, 15]. The codon adaptation index (CAI) [16] is an index of non-uniform codon use [17]. The CAI of each gene sequence of l codons is a measure of the bias in codon usage and it is defined as follows:

$$CAI = \left(\prod_{k=1}^{l} w_k \right)^{\frac{1}{l}} \; ; \quad CAI \in (0, 1) \tag{8}$$

where the product of $w_k = w(i_k)$ is considered over the DNA code under examination. The quantity $w(i_k)$ for a codon i at position k is given by the relative frequency of the codon i with respect to the most used codon for the same amino acid in a set of highly expressed genes (i.e. ribosomal proteins). In organisms the synonymous codons are not chosen randomly but they follow a rule, thuus it is more common that the beginning of the gene is more composed of rare codons. This is due to the influence of the machinery of translation that is conditioned by the presence of rare tRNAs [18, 19]. The

genes that present high values of CAI, for instance, ribosomal and major outer membrane genes whose products are required in large quantity with respect to other proteins for high duplication rates, are the effective fitness bottleneck of the bacterial duplication machinery. Until a few years ago, most of the researchers believed that the codon usage could be used to study the elongation rate in the translation process, being the pairing between codon and anticodon of the diffusing tRNA, a rate-limiting step compared with the peptide bond formation and translocation steps [20]. Recent discoveries based on ribosome profiling and next generation sequences seem to contrast the previous demonstration proving that rare codons, the so-called *slow codons*, are generally translated at similar average speed than abundant codons [21, 22, 23] . It remains *de-facto* that all the translational machines, and not only if we think to the duplication machinery, are dominated by regulation mechanisms. Furthermore, if the codon usage, in particular, the CAI, is not a *rate-limiting* index it does not affect the fact that a priori shows some new regularities in our multi-omic spaces and it is an oscillating omic on multi-omic patterns.

5.0.2 Transcriptomic layer: mRNA amounts

The omics extracted in the transcriptomic layer are the mRNA amounts and their fold changes (fc) caused to the effect of antibiotics. Two compendia of micro arrays are considered: The first compendium extracted from GEO with accession number GSE6836 is the one of Faith et al. [24] and contains 121 experimental conditions. The second compendia is of Suzuki et al. [25] with GEO accession number GSE59408 is based on the study E.coli resistance to 10 different antibiotics. From these compendia are selected 69 experiments between those one with a reasonable statistical reliability [26, 27] and a 'control vs treatment' expression matrix design. Reliabilities, described in equation 9, are fundamental in the definition of the Spearman's correct correlation (S. correction). The latter is leveraged for the compendia analysis within-studies and between-studies because is a stable form of correlation that take into account the noise propagation and stabilize the analyses considering microarray replicates. Then S. correction within-studies is considered on replicates of the same experiment in one of the possible compendia. S. correction between-studies is utilized when we compare mRNAs studies that come from different compendia between two different experiments (Figure 1 (red and blue arrows))) and in between-studies of the same exper-

iment but between replicates of controls and treatments (Figure 1 (green arrow)). Within-studies and between-studies median values of Spearman's correct correlations and Pearson's correlation (P.correction) are computed for each compendia. The results are reported in the Table 1. Once it is con-

Table 1: Median and standard deviation (±) within-studies (inter relation of all the i-th (i) microarray replicated measures within the control (C) or/and treatment (T)). Median and standard deviation (±) between-studies (cross relation between the replicated measures of the i-th (i) microarray and the j-th (j) microarray considering control (C) or/and treatment (T)). Median and standard deviation are above all Spearman's correction (*S.correction*) for attenuation and sample Pearson's correlation (*P.correlation*) in separate columns. (see also Figure 1)

	**Within Studies**			**Between Studies**	
1:			*Suzuki et al. (10 experiments)*		
	S. correction	P. correlation		S. correction	P. correlation
C_i/C_i	1		C_i/C_j	1	
T_i/T_i	0.97 ± 0.02	0.93 ± 0.03	T_i/T_j	0.98 ± 0.01	0.91 ± 0.02
C_i/T_i	0.95 ± 0.02	0.92 ± 0.03			
			CT_i/CT_j	0.99 ± 0.01	0.92 ± 0.02
2:			*Faith et al. (59 experiments)*		
	S. correction	P. correlation		S. correction	P. correlation
C_i/C_i	0.99 ± 0.01		C_i/C_j	1 ± 0.07	
T_i/T_i	1 ± 0.01	0.99 ± 0.01	T_i/T_j	0.98 ± 0.04	0.96 ± 0.04
C_i/T_i	0.92 ± 0.03	0.91 ± 0.03			
			CT_i/CT_j	1 ± 0.03	0.93 ± 0.04

sidered the Spearman's correct correlation through these data it is possible to prove that the selected experiments could be integrable and robust with respect the noise. After all the median values in Table 1 reports a maintained coherence within experiments reporting small variations between replicates of control and treatments. That's mean in specific contexts that antibiotics in the most of the cases considered presents a specific cellular target, thus altering the mRNA expression of a little group of genes (Table 1 C_i/T_is). The total mRNA extracted from the platforms is normalised to the averaged

value of $mRNAs/cell$ considering $\approx$ 3800 mRNA *copies/number* as scaling factor over all the compendia (see also supplementary material S1).

5.0.3 Proteomic layer: protein abundance and protein variation

E.*coli* steady-state protein abundance is downloaded from the PaxDb web-service [8]. We have considered all the studies with a coverage $\geq$ 98 % on the whole genome. In this way it is obtained the protein abundance in steady state. Then we need to extract the protein variation. The mRNAs amount and their relative fold changes play a central role in the evaluation of protein-level variation and, due to the heterogeneity of data, it is not a process so obvious. Furthermore, in the literature, there are a lot of methodologies for determining the variation of protein abundance caused by a treatment i.e. mRNAs levels, codon usage and amino-acid usage [28, 29]. In more published works mRNA and protein abundance are showed to be correlated weakly, with a coefficient of determination $R^2 \approx 0.17 - 0.47$ [30]. However, the lack of correlation it is imputed several control factors and due to the presence of noisy data [31]. It is possible to obtain a relevant correlation between protein abundance and mRNA if it is accounted the noise and separated from the rest of the information [27, 32]. Frost and Thompson [33] discussed several methods for the regression dilution bias. Then, protein variation will be inferred through a noise-robust linear model stabilized with reliabilities.

In the figure 2 the pipe-line of the method adopted is shown: steady-state protein abundance it is indicated as B and controls mRNAs amount as A. In this setting, the observations A and B are considered with additive noise. Then $\widetilde{A} = \alpha + n_\alpha$ with respectively $\widetilde{A} \approx N(\mu, \sigma_A^2)$ and noise $n_\alpha \approx N(0, \sigma_\alpha^2)$ with α the true value. Steady-steate protein abundance B are defined as the latter with $\widetilde{B} = \beta + n_\beta$. Consider $\widetilde{A}$ as a predictor of the mRNA controls true value α and $\widetilde{B}$ as the dependent variable. $\widetilde{A}$ and $\widetilde{B}$ are normally distributed. We are interested into the reliability of $\widetilde{A}$ that is defined as the variance ratio of the true value and the observed value:

$$\psi_\alpha = \frac{\sigma_\alpha^2}{\sigma_{\widetilde{A}}^2} = \frac{\sigma_\alpha^2}{\sigma_\alpha^2 + \sigma_{n_\alpha}^2} = \frac{\sigma_{\widetilde{A}}^2 - \sigma_{n_\alpha}^2}{\sigma_{\widetilde{A}}^2} \tag{9}$$

When the true value it is unknown the definition of reliability above remains theoretical. Even if it is possible to reach an estimation of ψ_α through the Pearson coefficient of correlation ρ. Then, each couple of observed values

(i,j) of $\widetilde{A}$ are considered as follows: $\widetilde{\psi_\alpha} = \rho_{\widetilde{A_i}\widetilde{A_j}}$. Reliability functions are furnished by Csardi et al. [27] in R. The reliability $\widetilde{\psi_\alpha}$ will be multiplied to an estimated slope according to some models in literature [34, 33]. Once the reliability it is obtained we can compute the protein variation leveraging a random effect model [35]. The random effect model considered is based on a linear regression with a random estimation (log-likelihood) for the slopes and intercepts. It is given as independent variable a selected group of mRNAs replicates in steady-state conditions (controls). The latter are chosen above all the 69 experiment considering those one that once perturbed are differentially expressed with a specific threshold γ and that maintain an acceptable reliability ($\psi_\alpha > 0.5$). The threshold γ permits to filter the differentially expressed genes by a variable fold change fc ($\pm fc$, $0.1 \leq fc \leq 1.2$) and a significant p-value (≤ 0.05 with Bonferroni correction). From these selected groups are removed the outliers. The predictors left are those biologically valid, because are those one that are really expected to change between experiments. Then, the latter could predict in a correct way the protein variation. If γ is more restrictive the reliability of A decrement making not affordable the predictors. In fact increasing the number of measurement, then the reliability coefficient increases:

$$\widetilde{\psi_\alpha^{new}} = \frac{\gamma \cdot \widetilde{\psi_\alpha}}{1 + (\gamma - 1) \cdot \widetilde{\psi_\alpha}} \tag{10}$$

where γ is the factor by which the test length is increased: it is proportionally related to the reliability. In a classical linear model for a j-th observation the mRNA predictors and the steady state protein abundance it is represented in the following form: $B_j = mA_j + \epsilon_j, \epsilon_j \approx N(0, \sigma^2)$ and $j = 1, ..., n$. Note that in this way it is not possible to consider the mRNA replicates decreasing the stability with respect the noise. Instead, the relationship in a random effect model it is repeated for N=8 controls times the replicates. Then the random effect model it is defined with $i = 1, ..., N$ and a shared variable θ_i as follows:

$$B_{ij} = \theta_i + mA_{ij} + \epsilon_{ij} \tag{11}$$

Due to the Wilks's theorem [36] we can compare, with a log likelihood ratio test, the null model $B_{ij} = mA_{ij} + \epsilon_{ij}$ (who does not take into account the random effect) with the random effect model $B_{ij} = \theta_i + mA_{ij} + \epsilon_{ij}$ [37, 32] (an approximated chi-square distribution with an associated p-value which

returns the goodness of fit). This procedure is repeated each time varying the fold change, thus obtaining a relevant fc threshold that could make the model significant.

Table 2: The table show the threshold utilized for obtaining a significant set of random effect model predictors used for inferring the protein variation. It is reported the log fold change in absolute value from 0.1 to 1.2, the $\widetilde{\psi_\alpha}$ reliabilities for what concerns the predictors and the log-likelihood test ratios between models approximated by a $\chi^2(3)$ distribution with a specific p-value.

	$\chi^2(3)$	χ^2 p-value	$\pm fc$	$\widetilde{\psi_\alpha}$
1	32.48	0.0000004151	0.1	0.699
2	32.48	0.0000004151	0.2	0.699
3	36.55	0.0000000572	0.3	0.691
4	16.77	0.0007890304	0.4	0.693
5	10.11	0.0176285375	0.5	0.687
6	5.65	0.1297964597	0.6	0.614
7	19.53	0.0002128110	0.7	0.529
8	11.38	0.0098197577	0.8	0.508
9	10.58	0.0142292459	0.9	0.486
10	8.74	0.0330070982	1	0.432
11	11.55	0.0090732111	1.1	0.417
12	10.42	0.0153329823	1.2	0.414

As showed in table 2 the optimal log fold change threshold is of 0.7 and it could be individuated at the seventh row of the table. At this threshold the random effect model says that mRNA control replicates (predictors) affect protein abundances (dependent variable) with a ($\chi^2(3) = 19.53, p = 0.00002$), increasing it by about $2.4e + 24$ molecules/cell $\pm 9.17e + 23$ (standard errors). Each θ_i will present its own slope and intercept then, it is possible to obtain a list of slopes and intercepts. Then, from this model, it is estimated a median intercept of $\hat{c} = -1.15e + 22$ and a median slope of $m_t = 2.98e + 24$. The latter, as said before, is corrected with its associated reliability ($\widetilde{\Psi}_\alpha = 0.529$) and become of $\hat{m} = 1.25e + 24$. Thus taking the parameters $\hat{m}$ and $\hat{c}$ we can have an estimation of the protein variation index pv_j that dimensionally is coherent with molecules/cell and is a function of the mRNA amounts x^t molecules/cell:

$$pv_j = \hat{m} \cdot x_j^t + \hat{c} \tag{12}$$

PaxDB's steady state protein abundance represented part per million (ppm) is transformed *molecules/cell* as described in the supplementary material S2.

5.0.4 Metabolomic layer: a novel integrated network

In this work the whole metabolic network is represented as a protein-centric network topology [38]. We have integrated in R the most updated E.coli metabolic network (furnished in supplementary material) from multiple sources: EcoCyc and KEGG. The network extracted from the Ecocyc smart tables is of $V=$ 1321 proteins and $E=$ 366778 reactions. Moreover, are integrated the KEGG pathways obtaining a network of $V=$ 1005 proteins and $E=$ 3394 edges. Ecocyc and KEGG networks are merged forming a network of $V=$ 1644 proteins and $E=$ 369863 edges. Each protein complex in the table of reactions is considered as a monomer by its individual reaction. Since we are interested on the relations of multi-omics projected on this structure we prove that this novel integrated network responds to the most well known topology properties present in literature.

It is studied the scale-free property of this network and we could asses, according to other studies [39, 40], that the integrated protein-centric metabolic network presents features of a power law degree (d) distribution Figure 3 with its typical parameter $\alpha = 2.7$. After that, the network topology is investigated across the features that characterise this metabolic network as a small world network: the clustering coefficient (CC) and the average path length (APL). In particular EcoCyc integrated network presents a $CC = 0.95$ and $APL = 2.26$, for what concern the novel metabolic network integrated (KEGG + EcoCyc) the $CC = 0.84$ and have an average path length of 2.71 proteins for shortest path. In both cases are presented some typical features of small-world networks: an high CC and a low APL [41]. Erdós-Rényi [42] models could be considered as a yardstick for deciding if we are dealing with a small-world network. In fact, it has been proven that real networks are small world networks, once compared to random networks, if presents the same number of edges and nodes, a similar ALP and a higher CC [43]. 100 Erdós-Rényi random networks of the same dimension of the integrated network are generated thus computing the CC median values and error (0.5103229 ± 0.0002) and the ALPs (1.86 ± 0.000005) with the conclusion that enjoys the small-world properties. If in one hand random networks are useful to describe the property of small-world between multi-omics pro-

jected on metabolic structures, on the other hand they not describe perfectly the real network under examination. In a certain sense, Poisson and exponential degree distribution could explain the evolution theory of proteins and compounds [38] because they randomly add on each epoch t new nodes to networks. Thus, some studies on E.*coli* based on these distributions account an average of 9.76 links per node [44]. In this setting, proteins are considered to accomplish the same charge of work not clearly explaining the complexity of the metabolic structures, because, for example, it is not well modeled the presence of hubs [45, 46]. Alternatively in scale-free networks is accounted a *preferential attachment* [43] that, in turn, show the high specificity of the enzyme and reactions and where it is possible to recognize hubs. It is important to underline that topology networks are helpful in describing the interaction between nodes but they do not describe the internal node characteristics.

6 S6 - MORA: toy example

Adjacency influences are considered on a toy model (figure 4 (a)). A network of nodes, named with a number from 1 to 13, is showed. The nodes are ordered following their relative positions forming this sequence: *3-1-2-4-5-6-7-8-10-11-13-9-12* (in pathways the positions are taken considering the distance by the origin of replication). According to the algorithm MORA, in the figure the nodes are showed with a more/less influence on the sequence (reciprocal influence) and their relative adjacency weights (more/less a node is influent, more/less its diameter increases on the network). For example, nodes *3-1-2* are adjacent and linked in the network showing relevant adjacent weights. Node 7 is the most influencing node on the sequence, due to the effect of its direct adjacency with nodes 6 and 8 on the sequence and its direct links to 6 and 8 on the network ($\psi = 2$). Furthermore, node 7 is involved in 2 specific shortest paths: in fact, the algorithm increases its weight, counting the influence effects of adjacent nodes 5-6 and 8-10 in the sequence, that on the network represent the shortest paths of three nodes (5-7-6) and (8-7-10) ($\psi = 3$) (node 7 is present in both). Influences are considered on undirected networks. Nodes with more/less influence are linked with their associated structural/reciprocal influence colour. Structural/reciprocal influence (value of $infl$) are divided and coloured forming the group of the most adjacent nodes (those with a weight $\geq$ median($infl$)) and that of the less adjacent nodes.(figure 4 (b) (c)). In these plots 2 extreme structural conditions are

tested with a sequence equal to *1-2-3-4-5*, where the first plot is a clique and the second a broken clique (snaked).

7 S7 - A concrete example for multi-omic metabolic network motifs: E.*coli* Glycolysis

In Figure 5 part (a) is shown an example of multi-omic oscillations on the pathway for the E.*coli* Glycolysis. Blue and red nodes show oscillating multi-omic values. Orange edges link nodes with the anti-dyadic effect (i.e. those with oscillating multi-omics), instead red and blue edges show the dyadic effect. In this case the anti-dyadic effect magnitude is of $\widehat{m_{01}} = 1.99$ and the dyadic effect magnitude of $\widehat{m_{1100}} = 2.14$. The different node sizes depict a less or more adjacent influences (computed with MORA). The reciprocal influence RI of Glycolysis with respect its sequence pattern is equal to 1. Then more adjacent nodes are those with adjacency weights greater than 1 and less adjacent nodes are those ≤ 1. Figure 5 part (b) shows multi-omic oscillations on patterns. The values are shown on a normalised scale and not yet binary discretized. The dots of several colours show a variation in oscillation due to the effect of the 69 considered treatments. The groups of operons and protein complexes that take part to the pathway are shown in the blue and red bands. The Glycolysis multi-omic pattern similarity to an ideal oscillating sequence is of $\sigma_{obs} = 0.62$. In Figure 6 part (a) are shown the multi-omic oscillations on the E.*coli* Glycolysis with path extensions. In this case, new reactions are added, and consequently also new nodes to the pathway and new multi-omics on the pattern. In this case, the anti-dyadic effect magnitude is $\widehat{m_{01}} = 1.35$ and the dyadic effect magnitude $\widehat{m_{00-11}} = 1.46$. With respect to figure 5 both the effects are lowered, but the network still maintains a significant amount of oscillating multi-omics ($\widehat{m_{01}} > 1$). In this case, the RI is $= 2$ showing that there are more adjacent influences with respect to the Glycolysis pathway without path extensions. In Figure 6 part (b) are shown multi-omic oscillations on patterns with the insertion of path extensions. With respect the standard conditions without modifications, where σ_{obs} was $= 0.6$, while now σ_{obs} is increased to 0.7413793. For analyzing the effect of treatments please refer to the tables of Additional File 1.

References

[1] Bartholomäus, A., Fedyunin, I., Feist, P., Sin, C., Zhang, G., Valleriani, A., Ignatova, Z.: Bacteria differently regulate mrna abundance to specifically respond to various stresses. Phil. Trans. R. Soc. A **374**(2063), 20150069 (2016)

[2] Milo, R., Jorgensen, P., Moran, U., Weber, G., Springer, M.: Bionumbers—the database of key numbers in molecular and cell biology. Nucleic acids research **38**(suppl 1), 750–753 (2010)

[3] Tenenbaum, D.: Keggrest: Client-side rest access to kegg. R package version **1**(1) (2013)

[4] Keseler, I.M., Bonavides-Martínez, C., Collado-Vides, J., Gama-Castro, S., Gunsalus, R.P., Johnson, D.A., Krummenacker, M., Nolan, L.M., Paley, S., Paulsen, I.T., *et al.*: Ecocyc: a comprehensive view of escherichia coli biology. Nucleic acids research **37**(suppl 1), 464–470 (2009)

[5] Kanehisa, M., Goto, S.: Kegg: kyoto encyclopedia of genes and genomes. Nucleic acids research **28**(1), 27–30 (2000)

[6] NCBI, R.C.: Database resources of the national center for biotechnology information. Nucleic acids research **41**(Database issue), 8 (2013)

[7] Barrett, T., Troup, D.B., Wilhite, S.E., Ledoux, P., Rudnev, D., Evangelista, C., Kim, I.F., Soboleva, A., Tomashevsky, M., Edgar, R.: Ncbi geo: mining tens of millions of expression profiles—database and tools update. Nucleic acids research **35**(suppl 1), 760–765 (2007)

[8] Wang, M., Herrmann, C.J., Simonovic, M., Szklarczyk, D., Mering, C.: Version 4.0 of paxdb: Protein abundance data, integrated across model organisms, tissues, and cell-lines. Proteomics **15**(18), 3163–3168 (2015)

[9] Davis, S., Meltzer, P.S.: Geoquery: a bridge between the gene expression omnibus (geo) and bioconductor. Bioinformatics **23**(14), 1846–1847 (2007)

[10] Smyth, G.K.: Limma: linear models for microarray data. In: Bioinformatics and Computational Biology Solutions Using R and Bioconductor, pp. 397–420. Springer, ??? (2005)

[11] Team, R.C., et al.: R: A language and environment for statistical computing (2013)

[12] Kleinberg, J., Tardos, E.: Algorithm Design. Pearson Education India, ??? (2006)

[13] Azar, Y., Broder, A.Z., Karlin, A.R., Upfal, E.: Balanced allocations. SIAM journal on computing **29**(1), 180–200 (1999)

[14] Chakraborty, S., Nag, D., Mazumder, T.H., Uddin, A.: Codon usage pattern and prediction of gene expression level in bungarus species. Gene **604**, 48–60 (2017)

[15] Miyasaka, H.: Translation initiation aug context varies with codon usage bias and gene length in drosophila melanogaster. Journal of molecular evolution **55**(1), 52–64 (2002)

[16] Sharp, P.M., Li, W.-H.: The codon adaptation index-a measure of directional synonymous codon usage bias, and its potential applications. Nucleic acids research **15**(3), 1281–1295 (1987)

[17] Komar, A.A.: The yin and yang of codon usage. Human Molecular Genetics, 207 (2016)

[18] Bentele, H.D.-P.K.: Mechanisms of translational regulation in bacteria: Impact on codon usage and operon organization. PhD thesis, Citeseer (2013)

[19] Gu, W., Zhou, T., Wilke, C.O.: A universal trend of reduced mrna stability near the translation-initiation site in prokaryotes and eukaryotes. PLoS Comput Biol **6**(2), 1000664 (2010)

[20] Grantham, R.L.: Codon usage in molecular evolution. eLS (2001)

[21] Ingolia, N.T., Lareau, L.F., Weissman, J.S.: Ribosome profiling of mouse embryonic stem cells reveals the complexity and dynamics of mammalian proteomes. Cell **147**(4), 789–802 (2011)

[22] Li, G.-W., Burkhardt, D., Gross, C., Weissman, J.S.: Quantifying absolute protein synthesis rates reveals principles underlying allocation of cellular resources. Cell **157**(3), 624–635 (2014)

[23] Li, G.-W., Oh, E., Weissman, J.S.: The anti-shine-dalgarno sequence drives translational pausing and codon choice in bacteria. Nature **484**(7395), 538–541 (2012)

[24] Faith, J.J., Hayete, B., Thaden, J.T., Mogno, I., Wierzbowski, J., Cottarel, G., Kasif, S., Collins, J.J., Gardner, T.S.: Large-scale mapping and validation of escherichia coli transcriptional regulation from a compendium of expression profiles. PLoS biol **5**(1), 8 (2007)

[25] Suzuki, S., Horinouchi, T., Furusawa, C.: Prediction of antibiotic resistance by gene expression profiles. Nature communications **5** (2014)

[26] Santos, J.R.A.: Cronbachs alpha: A tool for assessing the reliability of scales. Journal of extension **37**(2), 1–5 (1999)

[27] Csárdi, G., Franks, A., Choi, D.S., Airoldi, E.M., Drummond, D.A.: Accounting for experimental noise reveals that mrna levels, amplified by post-transcriptional processes, largely determine steady-state protein levels in yeast. PLoS Genet **11**(5), 1005206 (2015)

[28] Ingolia, N.T., Ghaemmaghami, S., Newman, J.R., Weissman, J.S.: Genome-wide analysis in vivo of translation with nucleotide resolution using ribosome profiling. science **324**(5924), 218–223 (2009)

[29] Li, J.J., Bickel, P.J., Biggin, M.D.: System wide analyses have underestimated protein abundances and the importance of transcription in mammals. PeerJ **2**, 270 (2014)

[30] Guimaraes, J.C., Rocha, M., Arkin, A.P.: Transcript level and sequence determinants of protein abundance and noise in escherichia coli. Nucleic acids research **42**(8), 4791–4799 (2014)

[31] Lu, P., Vogel, C., Wang, R., Yao, X., Marcotte, E.M.: Absolute protein expression profiling estimates the relative contributions of transcriptional and translational regulation. Nature biotechnology **25**(1), 117–124 (2007)

[32] Winter, B.: A very basic tutorial for performing linear mixed effects analyses. arXiv preprint arXiv:1308.5499 (2013)

[33] Frost, C., Thompson, S.G.: Correcting for regression dilution bias: comparison of methods for a single predictor variable. Journal of the Royal Statistical Society: Series A (Statistics in Society) **163**(2), 173–189 (2000)

[34] Fuller, W.A.: Measurement Error Models vol. 305. John Wiley & Sons, ??? (2009)

[35] Pinheiro, J., Bates, D., DebRoy, S., Sarkar, D.: R core team (2014) nlme: linear and nonlinear mixed effects models. r package version 3.1-117. Available at h ttp://CRAN. R-project. org/package= nlme (2014)

[36] Vuong, Q.H.: Likelihood ratio tests for model selection and non-nested hypotheses. Econometrica: Journal of the Econometric Society, 307–333 (1989)

[37] Winter, B.: Linear models and linear mixed effects models in r with linguistic applications. arXiv preprint arXiv:1308.5499 (2013)

[38] Light, S., Kraulis, P.: Network analysis of metabolic enzyme evolution in escherichia coli. Bmc Bioinformatics **5**(1), 1 (2004)

[39] Jeong, H., Tombor, B., Albert, R., Oltvai, Z.N., Barabási, A.-L.: The large-scale organization of metabolic networks. Nature **407**(6804), 651–654 (2000)

[40] Chung, F.R., Lu, L.: Complex Graphs and Networks vol. 107. American mathematical society Providence, ??? (2006)

[41] Altaf-Ul-Amin, M., Katsuragi, T., Sato, T., Kanaya, S.: A glimpse to background and characteristics of major molecular biological networks. Biomed Res Int **540297** (2015)

[42] Erd6s, P., Rényi, A.: On the evolution of random graphs. Publ. Math. Inst. Hungar. Acad. Sci **5**, 17–61 (1960)

[43] Albert, R., Barabási, A.-L.: Statistical mechanics of complex networks. Reviews of modern physics **74**(1), 47 (2002)

[44] Verkhedkar, K.D., Raman, K., Chandra, N.R., Vishveshwara, S.: Metabolome based reaction graphs of m. tuberculosis and m. leprae: a comparative network analysis. PLoS One **2**(9), 881 (2007)

[45] Barabási, A.-L., Frangos, J.: Linked: the New Science of Networks Science of Networks. Basic Books, ??? (2014)

[46] He, X., Zhang, J.: Why do hubs tend to be essential in protein networks? PLoS Genet **2**(6), 88 (2006)

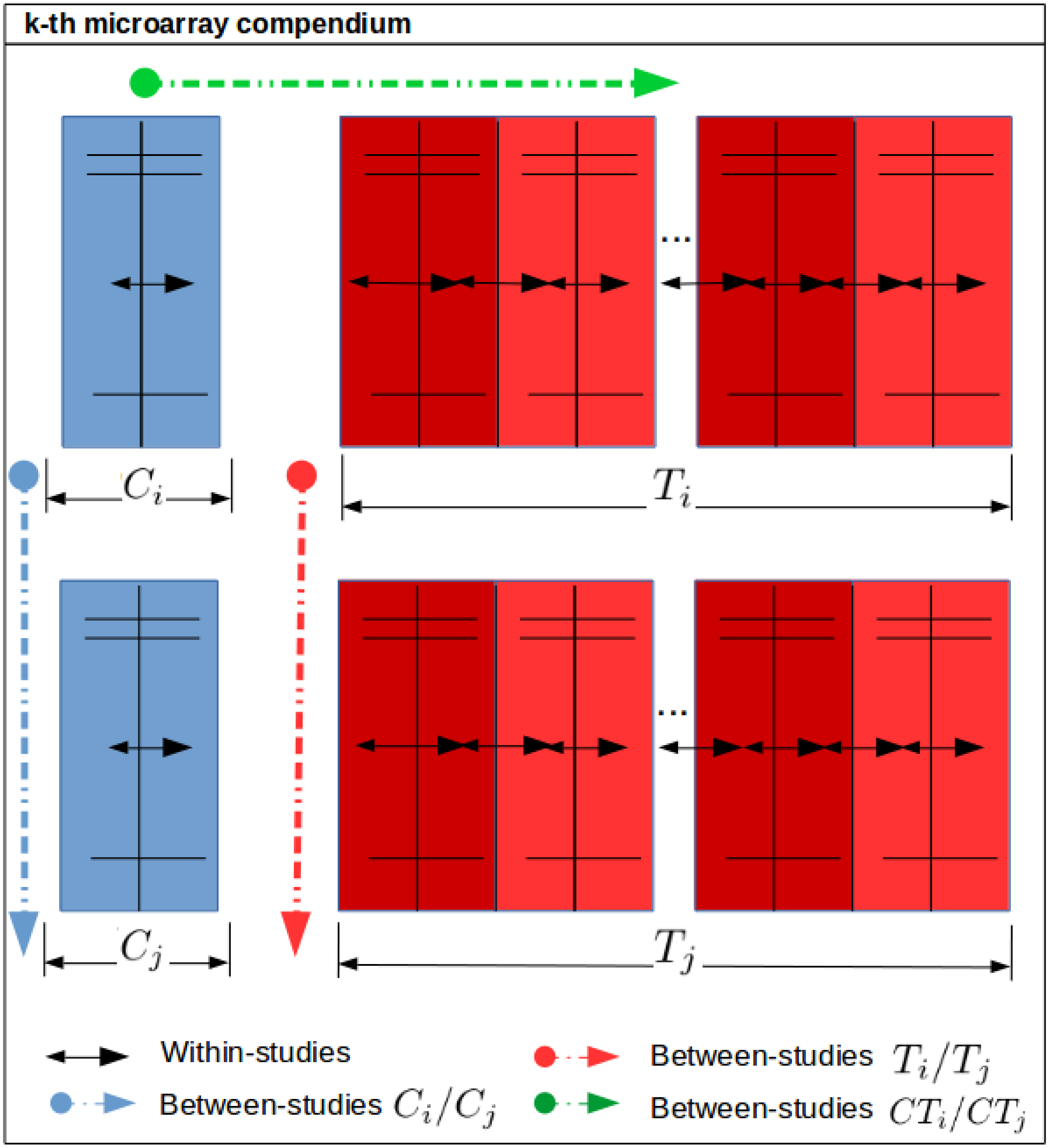

Figure 1: In figure 1 are reported the tables of control mRNA replicates (transcriptomic layer) in blue rectangles, while perturbed by treatments mRNA replicates in in red rectangles. C_i and C_j represent two distinct controls of the same k-th compendium. T_i and T_j represent two distinct treatments. Pearson's correlations and Spearman's corrected correlations are computed between-studies (blue, orange and red arrows) and within-studies (black arrows). Suzuki et al. experiments ($k = 1$) and Faith et al. ($k = 2$) experiments correlations are shown in the Table 1. Correlations are studied to show some characteristics of the microarray experiment design and to test the robustness of the dataset before executing other types of analysis.

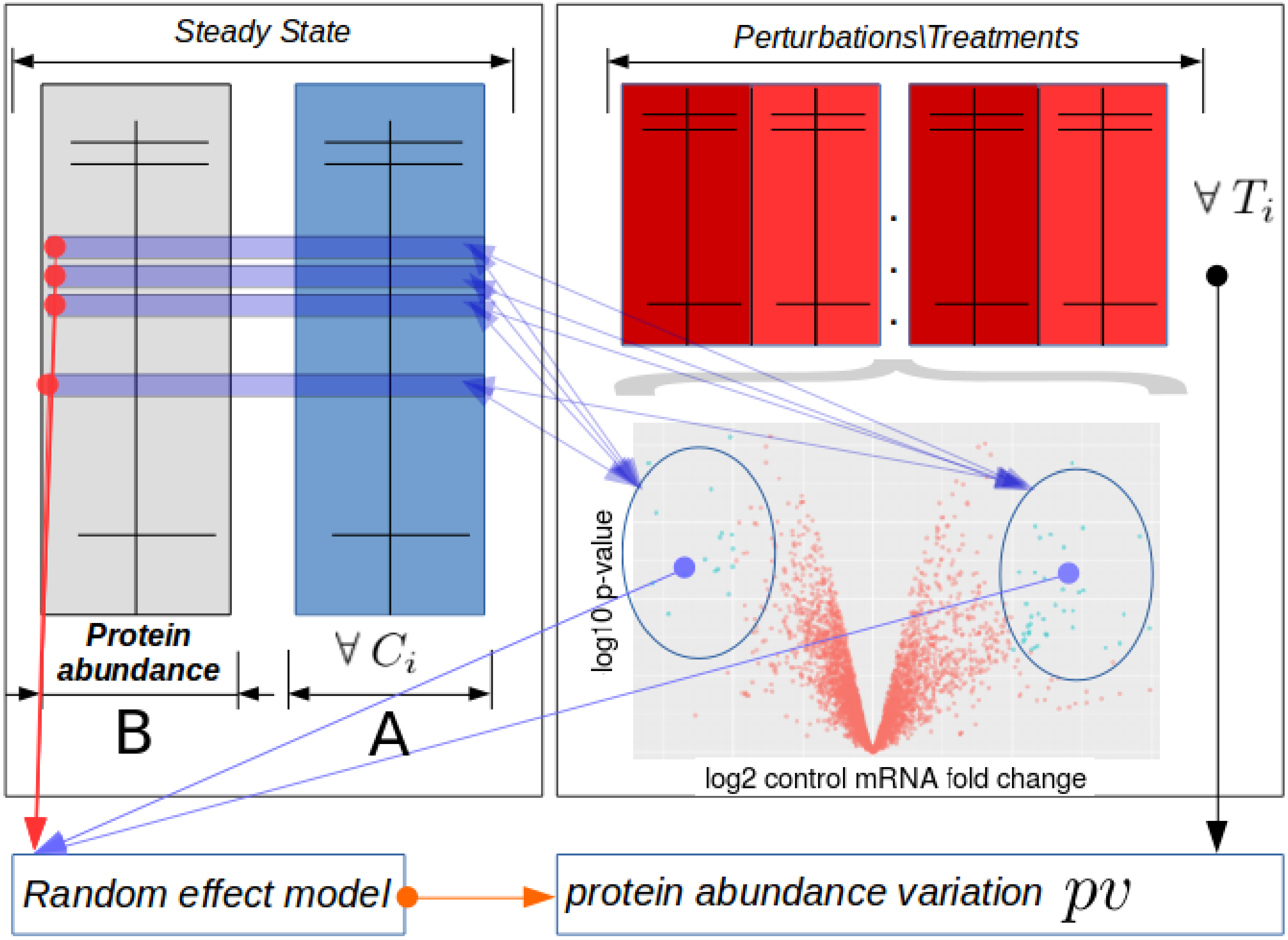

Figure 2: In this figure it is represented the pipeline that leads to a robust protein variation index (pv). The selected groups of differentially expressed genes are selected from each one of the 69 treatments (T_i - red rectangles). Then this set of predictors are utilized in the random effect model for the estimation of the slopes and the intercepts. The set of predictors (red and blue arrows) are extracted across all the groups of control mRNA amounts ($\forall C_i$) (blue rectangle A) and steady state protein abundances (grey rectangle B). This set is individuated above all the genes that presents an effective mRNA variation (volcano plot blue dots). Note that the set of predictors is computed between-studies (Suzuki et al. and Faith et al.) ($\forall T_i$) and leads to the estimation protein abundance variation for all the genes involved (see also 12).

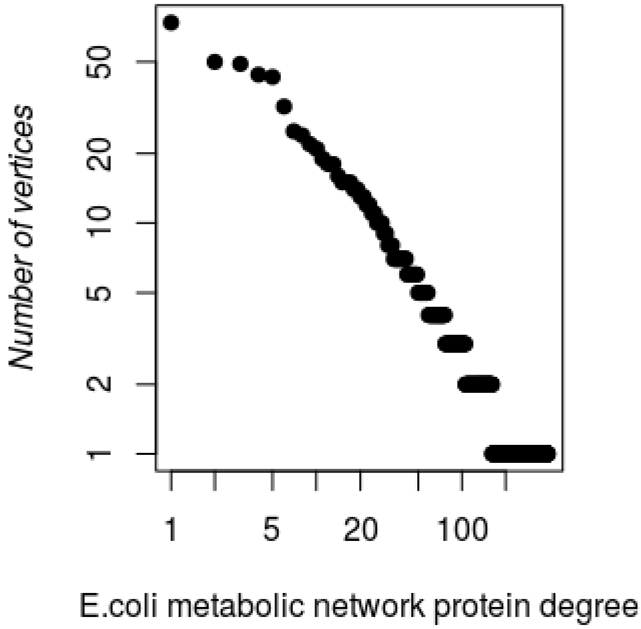

Figure 3: The plot shows the number of nodes and their degree (log-log scale) for the novel E.*coli* integrated metabolic network. The fitted power law degree with $\alpha = 2.7$. Average path length (APL) of 2.71 and an cluster coefficient (CC) of 0.84. The network shows typical characteristics of a metabolic network, where enzyme are strictly related each other (low APL) and in short circuits (high CC). Thus,the latter share substrate in input and output, transforming compounds, with high specificity.

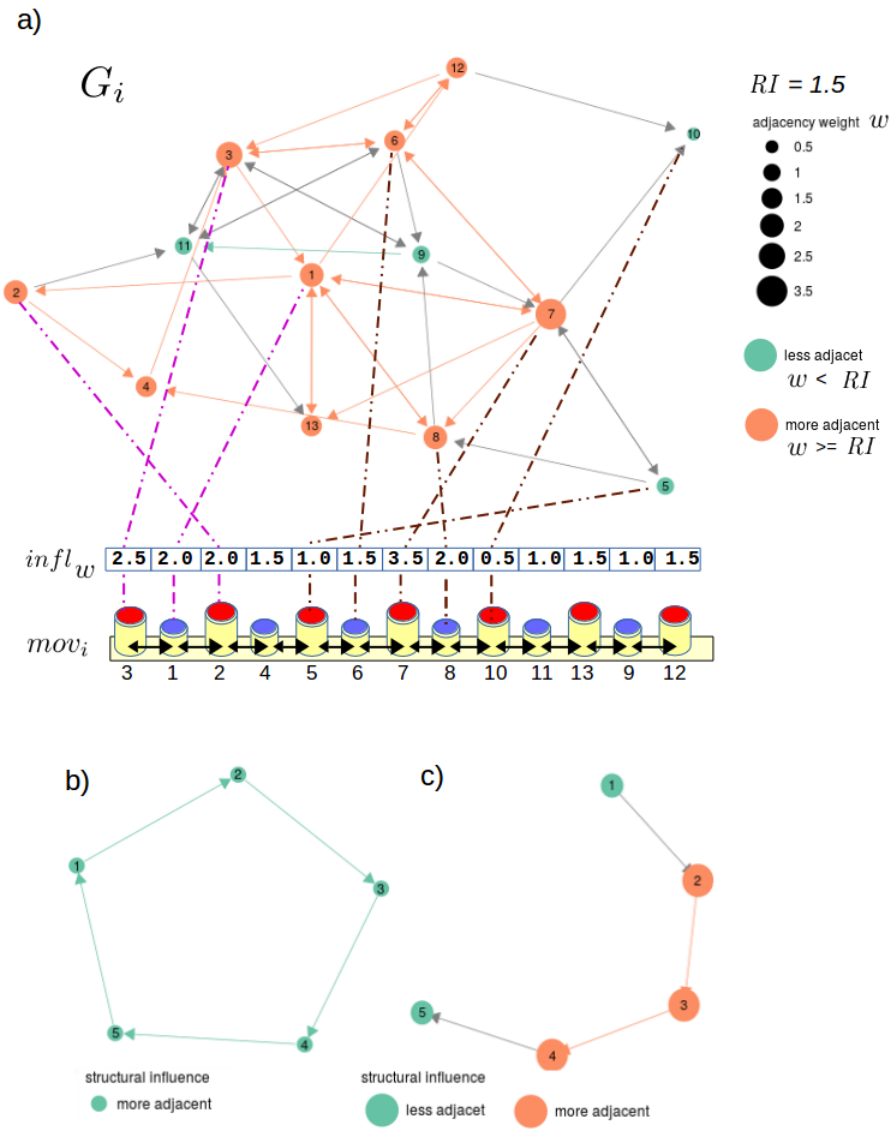

Figure 4: MORA: toy example. Please refer to section S6 of this file.

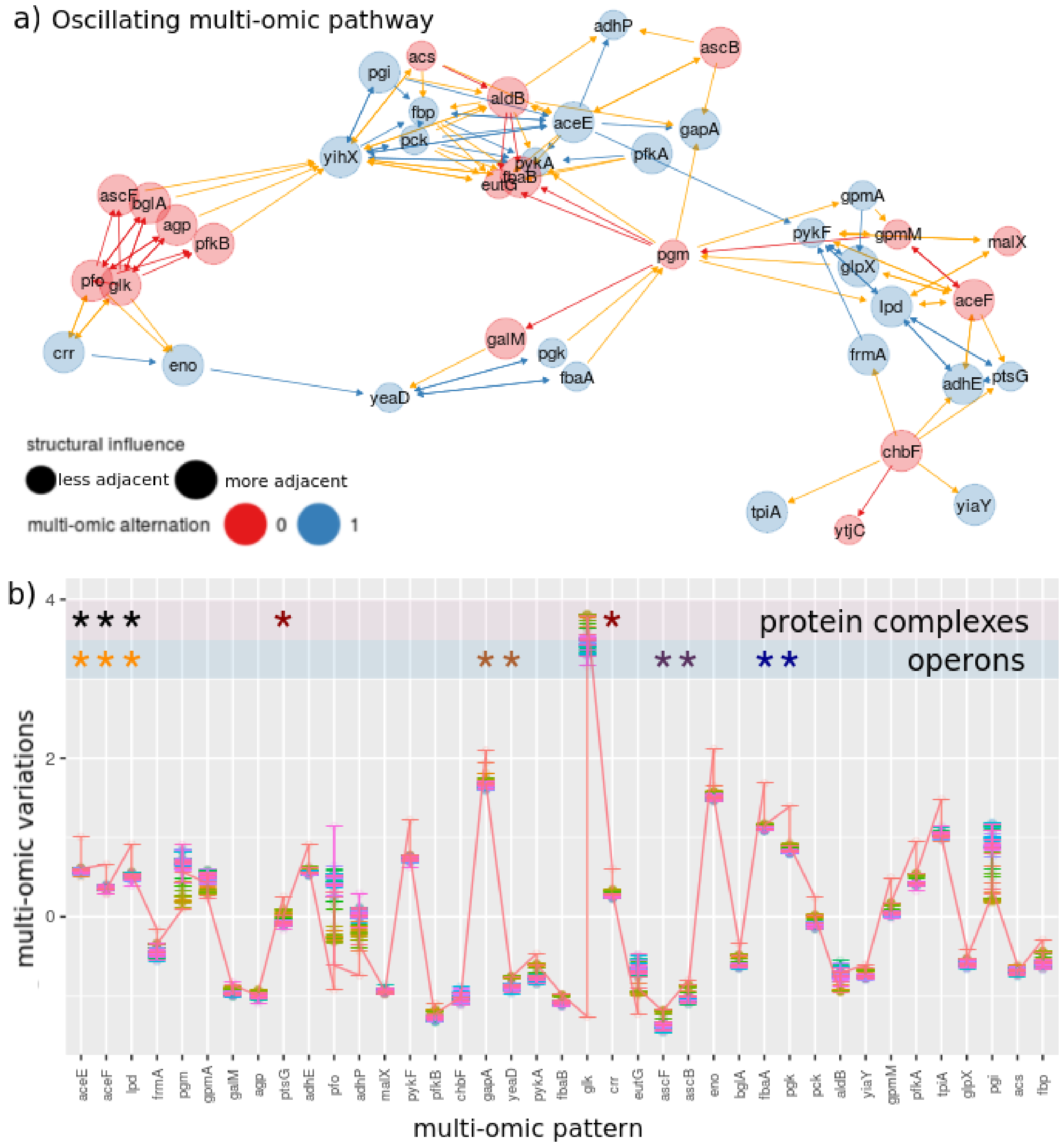

Figure 5: E.*coli* Glycolysis: multi-omic network motifs and patterns. Please refer to Section S7 of this file.

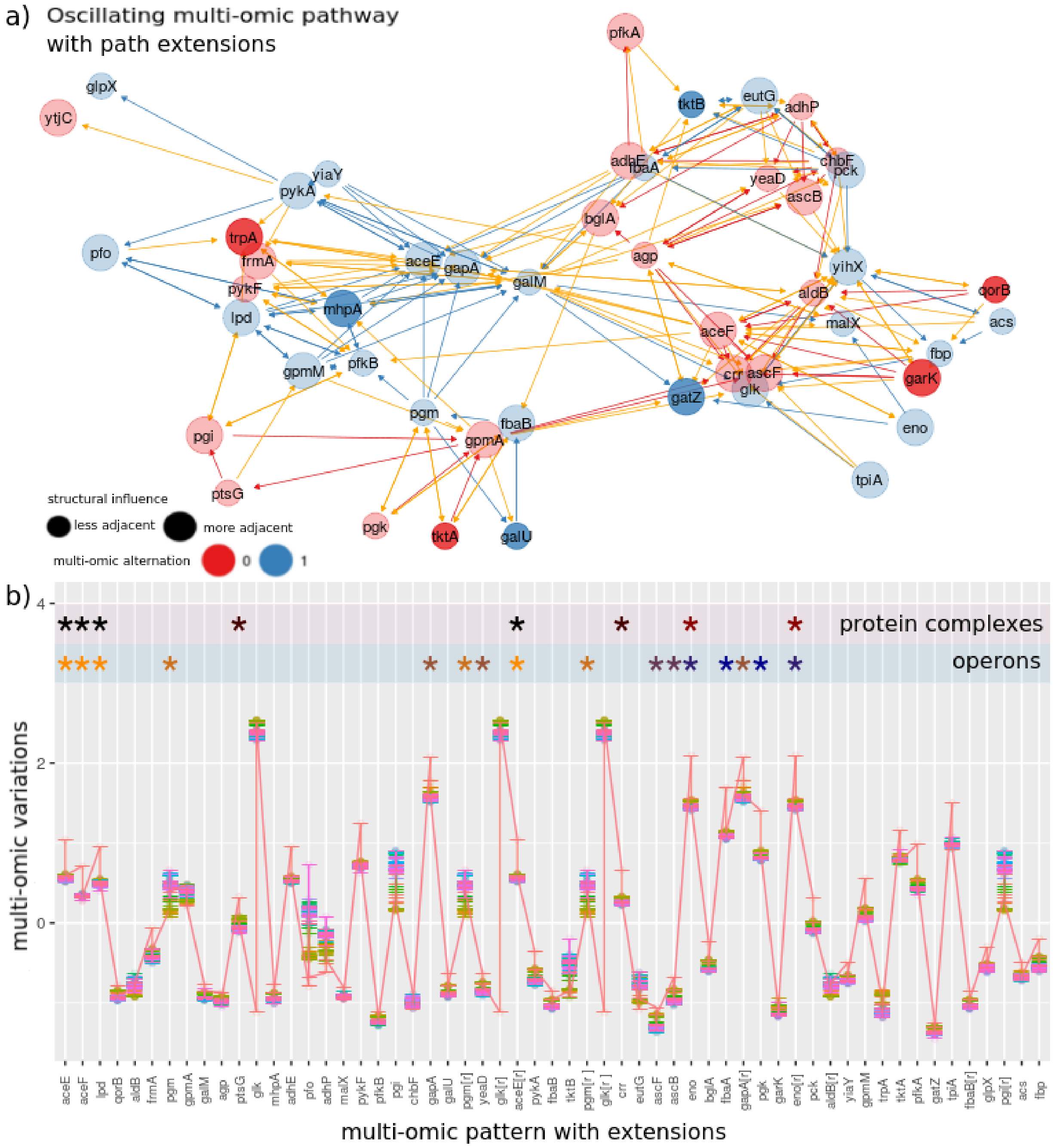

Figure 6: E.*coli* Glycolysis: multi-omic network motifs and patterns with extensions. Please refer to Section S7 of this file.
