# Supplementary material for: A study on multi-omic oscillations in Escherichia coli metabolic networks
Source: BMC Bioinformatics. 2018 Jul 9;19(Suppl 7):194. doi: 10.1186/s12859-018-2175-5 (PMC6069781; doi:10.1186/s12859-018-2175-5)
Supplement: Supplementary file 2 — It is a PDF document which contains 66 measures of dyadic/anti-dyadic effects, oscillation similarity scores, and reciprocal influences computed with MORA. Data are reported in standard conditions and considering the average effect of 69 treatments. (PDF 108 kb) [file 12859_2018_2175_MOESM2_ESM.pdf]

The study of multi-omic oscillations in Escherichia coli metabolic networks

Francesco Bardozzo
NeuRoNe Lab, DISA-MIS
Via Giovanni Paolo II 132
Salerno IT

Pietro Lió
Computer Laboratory
15 JJ Thomson Ave
Cambridge, UK

Roberto Tagliaferri
NeuRoNe Lab, DISA-MIS
Via Giovanni Paolo II 132
Salerno IT

November 8, 2017

1 Oscillating multi-omics measures on E.coli 66 pathways in standard conditions

The similarity of observed oscillating multi-omics patterns with respect to ideal patterns (σ_{obs}), the pathway dyadic/anti-dyadic effect magnitudes, $\widehat{m_{10}}$ and $\widehat{m_{1100}}$ and the reciprocal influences RI given in output from MORA, are observed on 66 pathways in standard conditions. These measures are taken on MLS without pathway modifications, with operon compressions, with path extensions and with operon compression followed by path extensions. Tables row names are labeled with with their unique E.*coli* KEGG identifier code ($eco : pathNNNNN$).

Steady state cond. MLS	Pathway				Pathway with path extensions				Pathway with operon compression				Pathway with operon compression and path extensions			
KEGG eco:path	σ_{obs}	$\widehat{m_{10}}$	$\widehat{m_{1100}}$	RI	σ_{obs}	$\widehat{m_{10}}$	$\widehat{m_{1100}}$	RI	σ_{obs}	$\widehat{m_{10}}$	$\widehat{m_{1100}}$	RI	σ_{obs}	$\widehat{m_{10}}$	$\widehat{m_{1100}}$	infl
00010	0.62	1.99	2.14	1.00	0.74	1.35	1.46	2.00	0.66	1.99	2.14	1.00	0.82	1.35	1.46	2.00
00020	0.38	2.40	1.40	1.50	0.68	0.91	1.13	2.00	0.62	2.40	1.40	1.50	0.69	0.91	1.13	4.50
00030	0.55	2.13	2.19	1.50	0.76	1.24	1.34	2.00	0.54	2.13	2.19	1.50	0.76	1.24	1.34	2.00
00040	0.67	2.56	1.38	1.50	0.76	1.78	1.37	2.00	0.47	2.56	1.38	1.50	0.67	1.78	1.37	2.00
00051	0.57	2.00	2.02	1.25	0.76	1.54	1.56	2.00	0.57	2.00	2.02	1.00	0.74	1.54	1.56	2.00
00052	0.72	1.94	2.06	1.00	0.76	1.44	1.59	1.50	0.65	1.94	2.06	1.00	0.62	1.44	1.59	1.50
00053	0.27	1.95	2.61	1.50	0.68	1.08	0.99	3.00	0.30	1.95	2.61	1.50	0.65	1.08	0.99	3.00
00061	0.64	1.08	1.74	1.25	0.64	0.39	1.52	1.50	0.57	1.08	1.74	1.00	0.67	0.39	1.52	1.50
00071	0.46	2.06	1.24	1.50	0.73	2.28	0.90	2.00	0.45	2.06	1.24	1.50	0.79	2.28	0.90	2.00
00130	0.58	2.16	2.45	1.00	0.68	0.84	0.72	3.50	0.69	2.16	2.45	1.00	0.68	0.84	0.72	3.25
00190	0.58	2.67	1.22	1.00	0.79	1.13	1.59	1.50	0.38	2.67	1.22	1.00	0.38	1.13	1.59	1.75
00220	0.75	2.05	2.04	1.50	0.87	1.39	1.36	2.00	0.71	2.05	2.04	1.50	0.86	1.39	1.36	2.00
00230	0.53	1.87	2.12	1.00	0.77	1.04	1.06	2.00	0.58	1.87	2.12	1.00	0.72	1.04	1.06	2.00
00240	0.52	1.76	2.11	1.00	0.80	1.40	1.16	2.00	0.53	1.76	2.11	1.00	0.75	1.40	1.16	2.00
00250	0.63	2.05	1.80	1.00	0.80	1.50	1.43	2.00	0.58	2.05	1.80	1.00	0.78	1.50	1.43	2.00
00260	0.69	1.73	2.29	1.00	0.72	1.39	1.77	1.50	0.71	1.73	2.29	1.00	0.71	1.39	1.77	1.50
00261	0.44	1.90	1.19	1.50	0.71	1.28	0.63	2.00	0.25	1.90	1.19	1.50	0.62	1.28	0.63	2.00
00270	0.53	1.63	2.13	1.00	0.67	1.22	1.54	2.00	0.57	1.63	2.13	1.00	0.71	1.22	1.54	2.00
00280	0.50	1.22	2.69	1.50	0.54	1.11	1.92	1.50	0.50	1.22	2.69	1.00	0.60	1.11	1.92	1.50
00290	0.43	1.67	2.36	1.50	0.47	1.35	1.97	1.50	0.29	1.67	2.36	1.00	0.40	1.35	1.97	1.50
00300	0.79	1.74	2.23	1.00	0.83	1.74	1.44	1.00	0.85	1.74	2.23	1.00	0.88	1.74	1.44	1.25
00310	0.64	1.47	1.72	1.50	0.75	1.73	1.63	1.50	0.38	1.47	1.72	1.00	0.56	1.73	1.63	1.50
00330	0.54	1.93	1.77	1.50	0.73	1.18	1.03	2.75	0.60	1.93	1.77	1.25	0.81	1.18	1.03	2.00
00340	0.29	1.00	3.36	1.50	0.73	1.36	1.34	2.00	0.29	1.00	3.36	1.50	0.75	1.36	1.34	2.00

00360	0.50	1.33	1.85	1.50	0.71	1.78	1.96	2.00	0.60	1.33	1.85	1.50	0.80	1.78	1.96	1.75
00362	0.33	1.82	2.52	2.00	0.50	0.98	1.31	3.50	0.60	1.82	2.52	1.75	0.78	0.98	1.31	2.75
00380	0.25	1.71	2.53	1.00	0.57	1.88	2.13	2.00	0.25	1.71	2.53	1.00	0.57	1.88	2.13	2.00
00400	0.70	1.82	1.75	1.00	0.78	1.54	1.51	1.50	0.47	1.82	1.75	1.25	0.60	1.54	1.51	1.50
00410	0.29	2.29	1.68	1.00	0.80	1.32	1.62	2.00	0.31	2.29	1.68	1.00	0.78	1.32	1.62	2.00
00440	0.75	1.67	1.67	1.50	0.75	1.67	1.67	1.50	0.75	1.67	1.67	1.50	0.75	1.67	1.67	1.50
00450	0.56	2.27	1.77	1.00	0.75	0.96	0.96	2.00	0.50	2.27	1.77	1.00	0.71	0.96	0.96	2.00
00480	0.72	2.29	1.67	1.50	0.74	1.69	1.29	1.50	0.72	2.29	1.67	1.50	0.74	1.69	1.29	1.50
00500	0.61	2.00	2.99	1.25	0.72	1.34	1.30	2.00	0.50	2.00	2.99	1.00	0.69	1.34	1.30	2.00
00520	0.57	2.21	1.81	1.00	0.68	1.25	1.20	2.00	0.54	2.21	1.81	1.00	0.66	1.25	1.20	2.00
00521	0.62	2.57	0.86	1.50	0.85	1.18	1.34	2.25	1.00	2.57	0.86	1.00	1.00	1.18	1.34	2.00
00540	0.54	1.82	1.77	1.00	0.73	0.97	0.77	2.50	0.48	1.82	1.77	1.00	0.71	0.97	0.77	2.50
00550	0.55	1.94	1.20	1.00	0.53	1.44	1.69	2.00	0.53	1.94	1.20	0.75	0.52	1.44	1.69	2.00
00561	0.73	2.20	2.46	1.00	0.71	1.82	2.70	1.50	0.60	2.20	2.46	1.00	0.62	1.82	2.70	1.50
00564	0.39	1.36	1.67	1.00	0.62	1.50	1.57	1.50	0.36	1.36	1.67	1.00	0.61	1.50	1.57	1.50
00620	0.38	1.98	2.06	1.50	0.76	0.79	0.86	4.00	0.45	1.98	2.06	1.50	0.82	0.79	0.86	4.00
00627	0.83	3.50	0.00	1.00	0.88	1.23	3.80	1.50	0.60	3.50	0.00	1.00	0.71	1.23	3.80	1.50
00630	0.47	1.89	1.81	1.00	0.73	1.21	1.27	2.00	0.46	1.89	1.81	1.00	0.77	1.21	1.27	2.00
00640	0.46	2.00	1.94	1.00	0.68	1.80	1.52	2.00	0.46	2.00	1.94	1.00	0.64	1.80	1.52	2.00
00650	0.38	1.59	2.00	1.00	0.65	0.82	0.91	2.00	0.48	1.59	2.00	1.00	0.63	0.82	0.91	3.75
00660	0.38	1.50	1.50	1.00	0.64	2.10	1.30	1.75	0.67	1.50	1.50	1.00	0.80	2.10	1.30	1.50
00670	0.38	1.71	2.33	1.75	0.72	1.36	1.89	2.00	0.38	1.71	2.33	1.75	0.72	1.36	1.89	2.00
00680	0.58	2.21	2.11	1.00	0.77	1.46	1.82	2.00	0.50	2.21	2.11	1.00	0.69	1.46	1.82	2.00
00730	0.57	1.91	2.08	1.00	0.74	1.68	1.49	1.50	0.57	1.91	2.08	1.00	0.74	1.68	1.49	1.50
00740	0.67	0.94	1.99	1.50	0.73	0.62	1.40	1.75	0.50	0.94	1.99	1.50	0.60	0.62	1.40	1.50
00750	0.50	1.92	2.32	1.50	0.62	0.95	1.51	2.00	0.57	1.92	2.32	1.25	0.67	0.95	1.51	2.00
00760	0.48	1.94	2.31	1.50	0.69	1.65	1.54	2.00	0.50	1.94	2.31	1.50	0.67	1.65	1.54	2.00
00770	0.48	1.81	2.05	1.50	0.69	1.08	1.96	2.00	0.50	1.81	2.05	1.50	0.64	1.08	1.96	2.00

00780	0.38	1.45	5.17	1.00	0.52	1.12	2.60	2.00	0.56	1.45	5.17	1.00	0.50	1.12	2.60	1.50
00790	0.43	1.65	2.54	1.00	0.50	0.76	1.27	2.00	0.39	1.65	2.54	1.00	0.48	0.76	1.27	2.50
00860	0.52	2.19	1.34	1.50	0.67	1.83	1.48	2.00	0.63	2.19	1.34	1.25	0.76	1.83	1.48	2.00
00900	0.33	1.53	2.68	0.50	0.61	0.49	0.79	4.00	0.40	1.53	2.68	0.50	0.62	0.49	0.79	3.00
00910	0.41	1.66	2.50	1.00	0.71	0.65	1.26	3.50	0.50	1.66	2.50	1.50	0.54	0.65	1.26	3.50
00970	0.71	0.80	5.33	1.00	0.77	1.22	2.82	1.25	0.62	0.80	5.33	1.00	0.72	1.22	2.82	1.50
02020	0.49	1.54	1.87	1.00	0.60	1.36	1.45	1.50	0.57	1.54	1.87	1.00	0.60	1.36	1.45	1.00
02030	0.53	1.78	1.91	1.25	0.69	1.11	1.26	2.00	0.60	1.78	1.91	1.00	0.83	1.11	1.26	1.50
02040	0.43	2.50	1.25	1.00	0.73	1.83	1.47	1.50	0.50	2.50	1.25	0.50	0.58	1.83	1.47	2.00
02060	0.65	1.84	1.64	1.00	0.79	1.62	1.34	1.50	0.67	1.84	1.64	1.00	0.68	1.62	1.34	1.50
03018	1.00	3.50	0.00	1.00	1.00	3.50	0.00	1.00	1.00	3.50	0.00	1.00	1.00	3.50	0.00	1.00
03410	0.73	0.00	2.36	1.00	0.83	2.40	2.61	1.00	0.73	0.00	2.36	1.00	0.83	2.40	2.61	1.00
03430	0.69	0.00	2.47	1.00	0.89	1.87	2.11	1.00	0.69	0.00	2.47	1.00	0.89	1.87	2.11	1.00
04122	0.53	1.79	2.69	1.00	0.61	1.65	2.55	1.50	0.50	1.79	2.69	1.00	0.60	1.65	2.55	1.00

Table 1:

2 Oscillating multi-omics measures on E.coli 66 pathways taking into account the average effect of 69 antibiotic treatments

The similarity of observed oscillating multi-omics patterns with respect to ideal patterns (σ_{obs}), the pathway dyadic/anti-dyadic effect magnitudes, $\widehat{m_{10}}$ and $\widehat{m_{1100}}$ and the reciprocal influences RI given in output from MORA, are observed on 66 pathways taking into account the average effect of 69 integrated perturbations induced by antibiotics. These measures are taken on MLS without pathway modifications, with operon compressions, with path extensions and with operon compression followed by path extensions. Tables S1 and S2 row names are labeled with theirs unique E.*coli* KEGG identifier code ($eco : pathNNNNN$).

Perturbed (69 treatments) MLS	Pathway				Pathway path extensions				Pathway operon compression				Pathway operon compression and path extensions			
KEGG eco:path	σ_{obs}	$\widehat{m_{10}}$	$\widehat{m_{1100}}$	infl	σ_{obs}	$\widehat{m_{10}}$	$\widehat{m_{1100}}$	infl	σ_{obs}	$\widehat{m_{10}}$	$\widehat{m_{1100}}$	infl	σ_{obs}	$\widehat{m_{10}}$	$\widehat{m_{1100}}$	infl
00010	0.50	1.81	1.88	1.00	0.59	1.30	1.50	2.00	0.58	1.81	1.88	1.00	0.72	1.30	1.50	1.50
00020	0.58	1.74	1.89	1.50	0.55	0.58	0.92	2.00	0.61	1.74	1.89	1.50	0.48	0.58	0.92	2.00
00030	0.71	1.85	2.17	1.50	0.63	1.08	1.30	2.00	0.72	1.85	2.17	1.50	0.78	1.08	1.30	2.00
00040	0.54	1.84	2.20	1.50	0.41	1.52	1.68	2.00	0.53	1.84	2.20	1.50	0.57	1.52	1.68	2.00
00051	0.58	1.80	2.14	1.25	0.72	1.53	1.51	2.00	0.63	1.80	2.14	1.00	0.60	1.53	1.51	2.00
00052	0.51	1.83	2.74	1.00	0.47	1.35	1.81	2.00	0.56	1.83	2.74	1.00	0.52	1.35	1.81	1.50
00053	0.39	2.99	0.80	1.50	0.42	0.87	1.12	3.00	0.23	2.99	0.80	1.50	0.43	0.87	1.12	2.00
00061	0.36	1.59	1.11	1.25	0.64	1.07	1.40	1.50	0.14	1.59	1.11	1.00	0.50	1.07	1.40	1.50
00071	0.75	2.59	1.35	1.50	0.70	2.37	1.27	2.00	0.60	2.59	1.35	1.50	0.78	2.37	1.27	2.00
00130	0.63	2.07	3.74	1.00	0.38	0.90	1.05	3.50	0.69	2.07	3.74	1.00	0.39	0.90	1.05	2.00
00190	0.62	1.44	1.79	1.00	0.78	1.10	1.37	1.50	0.50	1.44	1.79	1.00	0.58	1.10	1.37	1.75
00220	0.73	1.71	2.49	1.50	0.72	1.32	1.97	2.00	0.69	1.71	2.49	1.50	0.74	1.32	1.97	2.00
00230	0.54	2.01	1.89	1.00	0.52	1.09	0.97	2.00	0.62	2.01	1.89	1.00	0.64	1.09	0.97	2.00
00240	0.58	2.16	1.91	1.00	0.58	1.35	1.19	2.00	0.64	2.16	1.91	1.00	0.66	1.35	1.19	2.00
00250	0.63	2.05	1.74	1.00	0.68	1.77	1.14	2.00	0.62	2.05	1.74	1.00	0.71	1.77	1.14	2.00
00260	0.50	1.94	2.13	1.00	0.61	1.58	1.92	1.50	0.53	1.94	2.13	1.00	0.54	1.58	1.92	1.50
00261	0.68	2.31	1.53	1.50	0.72	1.39	0.59	2.00	0.49	2.31	1.53	1.50	0.65	1.39	0.59	2.00
00270	0.53	1.95	2.31	1.00	0.56	1.33	1.94	2.00	0.57	1.95	2.31	1.00	0.69	1.33	1.94	2.00
00280	0.67	2.10	1.88	1.50	0.55	2.21	1.30	1.50	0.71	2.10	1.88	1.25	0.56	2.21	1.30	1.50
00290	0.52	1.69	2.05	1.50	0.65	1.37	1.77	1.50	0.46	1.69	2.05	1.00	0.51	1.37	1.77	1.50
00300	0.38	2.19	1.62	1.00	0.47	1.71	1.41	1.00	0.42	2.19	1.62	1.00	0.43	1.71	1.41	1.00
00310	0.30	1.55	2.52	1.50	0.64	1.30	2.10	1.50	0.43	1.55	2.52	1.00	0.71	1.30	2.10	1.50
00330	0.69	2.06	1.88	1.50	0.49	1.05	1.18	2.75	0.80	2.06	1.88	1.25	0.65	1.05	1.18	2.00

00340	0.32	1.42		1.50	0.45	2.37	0.44	2.00	0.32	1.42		1.50	0.75	2.37	0.44	2.00
00360	0.60	1.73	2.15	1.50	0.56	1.73	1.86	2.00	0.65	1.73	2.15	1.50	0.58	1.73	1.86	1.50
00362	0.39	2.24	1.58	2.00	0.50	1.44	0.81	2.50	0.24	2.24	1.58	1.50	0.44	1.44	0.81	2.00
00380	0.29	2.24	1.12	1.25	0.62	2.17	1.88	2.00	0.29	2.24	1.12	1.25	0.62	2.17	1.88	2.00
00400	0.75	1.86	1.79	1.00	0.70	1.56	1.58	1.50	0.53	1.86	1.79	1.25	0.56	1.56	1.58	1.50
00410	0.50	1.96	2.57	1.00	0.60	1.06	2.26	2.00	0.38	1.96	2.57	1.00	0.65	1.06	2.26	2.00
00440	0.75	1.67		1.50	0.75	1.67		1.50	0.75	1.67		1.50	0.75	1.67		1.50
00450	0.71	2.21	1.81	1.00	0.62	1.12	0.99	2.00	0.76	2.21	1.81	1.00	0.57	1.12	0.99	2.00
00480	0.67	2.46	2.53	1.50	0.68	1.77	1.43	1.50	0.67	2.46	2.53	1.50	0.58	1.77	1.43	1.50
00500	0.67	1.89	2.60	1.00	0.48	1.29	1.38	2.00	0.72	1.89	2.60	1.00	0.61	1.29	1.38	2.00
00520	0.49	1.88	1.93	1.00	0.58	1.42	1.03	2.00	0.55	1.88	1.93	1.00	0.65	1.42	1.03	2.00
00521	0.50	1.93	2.01	1.50	0.69	1.31	1.08	2.25	0.50	1.93	2.01	1.00	0.80	1.31	1.08	1.75
00540	0.52	1.93	1.92	1.00	0.38	1.01	0.73	2.50	0.55	1.93	1.92	1.00	0.59	1.01	0.73	2.00
00550	0.64	1.36	2.65	1.00	0.46	1.23	1.57	2.00	0.53	1.36	2.65	0.75	0.50	1.23	1.57	1.25
00561	0.39	1.73	3.32	1.00	0.50	1.77	2.52	1.50	0.41	1.73	3.32	1.00	0.50	1.77	2.52	1.50
00564	0.50	2.36	1.61	1.00	0.49	1.74	1.40	1.50	0.48	2.36	1.61	1.00	0.48	1.74	1.40	1.50
00620	0.53	2.06	2.11	1.50	0.49	0.73	0.87	4.00	0.59	2.06	2.11	1.50	0.56	0.73	0.87	2.00
00627	0.48	3.96		1.00	0.73	1.34	6.79	1.50	0.30	3.96		1.00	0.55	1.34	6.79	1.50
00630	0.55	1.81	2.19	1.00	0.60	1.20	1.32	2.00	0.48	1.81	2.19	1.00	0.70	1.20	1.32	2.00
00640	0.30	2.20	1.75	1.00	0.36	1.83	1.65	2.00	0.29	2.20	1.75	1.00	0.40	1.83	1.65	2.00
00650	0.53	2.07	1.91	1.00	0.50	0.75	1.06	2.00	0.52	2.07	1.91	1.00	0.48	0.75	1.06	2.00
00660	0.31	1.40	1.39	1.00	0.41	1.24	1.16	1.75	0.66	1.40	1.39	1.00	0.40	1.24	1.16	1.50
00670	0.47	1.90	2.08	1.75	0.45	1.45	1.75	2.00	0.47	1.90	2.08	1.75	0.51	1.45	1.75	2.00
00680	0.38	1.79	2.14	1.00	0.62	1.55	1.83	2.00	0.28	1.79	2.14	0.75	0.54	1.55	1.83	2.00
00730	0.80	1.46	2.48	1.00	0.54	1.21	2.07	1.50	0.80	1.46	2.48	1.00	0.57	1.21	2.07	1.50
00740	0.50	1.29	3.17	1.50	0.55	0.82	1.78	2.00	0.57	1.29	3.17	1.50	0.57	0.82	1.78	1.50
00750	0.63	1.87	1.28	1.50	0.69	1.26	1.46	2.00	0.71	1.87	1.28	1.25	0.70	1.26	1.46	2.00
00760	0.76	1.71	2.09	1.50	0.57	1.52	1.64	2.00	0.78	1.71	2.09	1.50	0.61	1.52	1.64	2.00

00770	0.60	1.64	2.25	1.50	0.64	1.47	2.22	2.00	0.53	1.64	2.25	1.50	0.52	1.47	2.22	2.00
00780	0.25	1.41	4.51	1.00	0.58	1.15	2.30	2.00	0.38	1.41	4.51	1.00	0.43	1.15	2.30	1.25
00790	0.67	2.19	1.74	1.00	0.47	1.17	1.15	2.00	0.72	2.19	1.74	1.00	0.65	1.17	1.15	2.00
00860	0.48	1.68	2.29	1.50	0.48	1.49	1.73	2.00	0.42	1.68	2.29	1.25	0.54	1.49	1.73	2.00
00900	0.55	1.71	2.11	0.75	0.39	0.76	0.68	4.00	0.67	1.71	2.11	0.75	0.60	0.76	0.68	2.00
00910	0.55	2.06	2.02	1.25	0.55	0.62	0.92	3.50	0.59	2.06	2.02	1.50	0.59	0.62	0.92	3.50
00970	0.54	3.26	1.68	1.00	0.54	1.81	2.80	1.25	0.52	3.26	1.68	1.00	0.57	1.81	2.80	1.50
02020	0.45	1.89	1.85	1.00	0.44	1.50	1.94	1.50	0.52	1.89	1.85	1.00	0.49	1.50	1.94	1.00
02030	0.26	0.60	1.50	1.25	0.42	0.99	0.92	2.00	0.50	0.60	1.50	1.00	0.60	0.99	0.92	1.00
02040	0.46	1.94	1.46	1.00	0.55	2.19	1.32	1.50	0.50	1.94	1.46	0.50	0.69	2.19	1.32	1.75
02060	0.44	2.16	2.58	1.00	0.53	1.65	1.44	1.50	0.58	2.16	2.58	1.00	0.63	1.65	1.44	1.00
03018	0.86	3.50	0.00	1.00	0.86	3.50	0.00	1.00	0.86	3.50	0.00	1.00	0.86	3.50	0.00	1.00
03410	0.63	0.00	3.13	1.00	0.62	2.57	2.52	1.00	0.63	0.00	3.13	1.00	0.62	2.57	2.52	1.00
03430	0.47	0.00	2.90	1.00	0.58	2.16	1.59	1.00	0.47	0.00	2.90	1.00	0.61	2.16	1.59	1.00
04122	0.60	2.27	1.95	1.00	0.44	1.96	1.59	1.50	0.58	2.27	1.95	1.00	0.42	1.96	1.59	1.00

Table 2:
